# An Online Randomized Controlled Trial Evaluating HIV Prevention Digital Media Interventions for Men Who Have Sex with Men

**DOI:** 10.1371/journal.pone.0046252

**Published:** 2012-10-02

**Authors:** Sabina Hirshfield, Mary Ann Chiasson, Heather Joseph, Roberta Scheinmann, Wayne D. Johnson, Robert H. Remien, Francine Shuchat Shaw, Reed Emmons, Gary Yu, Andrew D. Margolis

**Affiliations:** 1 Public Health Solutions, New York, New York, United States of America; 2 Division of HIV/AIDS Prevention, Prevention Research Branch, National Center for HIV, Viral Hepatitis, STD, and TB Prevention, Centers for Disease Control and Prevention, Atlanta, Georgia, United States of America; 3 HIV Center for Clinical and Behavioral Studies, New York State Psychiatric Institute, Columbia University, New York, New York, United States of America; 4 Steinhardt School of Culture, Education, and Human Development, New York University, New York, New York, United States of America; 5 Department of Biostatistics, Mailman School of Public Health, Columbia University, New York, New York, United States of America; University of Ottawa, Canada

## Abstract

**Background:**

As HIV infection continues unabated, there is a need for effective interventions targeting at-risk men who have sex with men (MSM). Engaging MSM online where they meet sexual partners is critical for HIV prevention efforts.

**Methods:**

A randomized controlled trial (RCT) conducted online among U.S. MSM recruited from several gay sexual networking websites assessed the impact of 2 HIV prevention videos and an HIV prevention webpage compared to a control condition for the study outcomes HIV testing, serostatus disclosure, and unprotected anal intercourse (UAI) at 60-day follow-up. Video conditions were pooled due to reduced power from low retention (53%, n = 1,631). No participant incentives were provided.

**Principal Findings:**

Follow-up was completed by 1,631 (53%) of 3,092 eligible men. In the 60 days after the intervention, men in the pooled video condition were significantly more likely than men in the control to report full serostatus disclosure (‘asked and told’) with their last sexual partner (OR 1.32, 95% CI 1.01–1.74). Comparing baseline to follow-up, HIV-negative men in the pooled video (OR 0.70, 95% CI 0.54–0.91) and webpage condition (OR 0.43, 95% CI 0.25–0.72) significantly reduced UAI at follow-up. HIV-positive men in the pooled video condition significantly reduced UAI (OR 0.38, 95% CI 0.20–0.67) and serodiscordant UAI (OR 0.53, 95% CI 0.28–0.96) at follow-up.

**Conclusions/Significance:**

Findings from this online RCT of MSM recruited from sexual networking websites suggest that a low cost, brief digital media intervention designed to engage critical thinking can increase HIV disclosure to sexual partners and decrease sexual risk. Effective, brief HIV prevention interventions featuring digital media that are made widely available may serve as a complementary part of an overall behavioral and biomedical strategy for reducing sexual risk by addressing the specific needs and circumstances of the target population, and by changing individual knowledge, motivations, and community norms.

**Trial Registration:**

ClinicalTrials.gov NCT00649701

## Introduction

Only an estimated 19% of the more than 1 million HIV-infected persons in the U.S have an undetectable viral load, [1] which is likely the result of social and behavioral barriers, [2] including the lack of awareness of HIV status. [3] It is therefore not surprising that overall HIV infection rates have been relatively stable in the U.S., though men who have sex with men (MSM) remain disproportionately affected by the transmission of HIV. [4] New HIV infections in MSM have been attributed in part to increased access to sex partners via online meeting websites, [5] thereby increasing the potential for transmission of HIV and other sexually transmitted infections (STIs). [6] HIV prevention studies conducted online have found that men who meet men online report more sex partners, casual partners, [7], [8] and unprotected anal intercourse (UAI) than do men who meet partners offline. [9] It is therefore critical to deliver behavioral interventions to MSM online to reach and engage men where they meet sex partners [10], [11], [12].

Early in the epidemic, community-wide safer sex messages delivered through print ads and media campaigns were effective at educating the gay community and lowering transmission risk behaviors. [13], [14] Since then, effective HIV prevention interventions geared toward changing high-risk behavior have been developed, [15] though many of these interventions have been in small-group formats, [16] which tend to be costly (i.e., labor-intensive, require many trained professionals), difficult to implement and sustain, and lack large-scale reproducibility. [17], [18] Thus, online behavioral interventions, particularly low intensity methods, hold much promise for the future of HIV prevention as part of a multifaceted approach to risk reduction, given their relatively low cost to implement and potential to reach a wide audience of at-risk persons efficiently. [17], [19].

Technology-based HIV behavioral interventions are increasingly incorporating digital media, ranging from brief, untailored video interventions to complex computer-tailored multimedia interventions that target individual behaviors. [20], [21] As an intervention tool, digital media can be delivered in a variety of settings, such as in schools or clinics, [22] and can be utilized electronically via text messaging, [23] handheld computers or Smartphones, [24] or online. [25], [26], [27], [28] Videos are a prime example of using digital media to engage learners, and they have been used as an HIV prevention tool since the 1980s. [29] Further, online video-based interventions are an appealing and effective way to deliver HIV prevention content to MSM. [27], [28], [30] They can be easily replicated after development, require minimal staffing, have broad geographic reach, and have the potential to change both community norms and individual behavior at low cost. [31].

Although online research studies tend to report higher attrition than offline research as there are fewer social constraints, [32] a growing number of validity studies indicate higher reporting of sexual risk and substance-using behaviors with computer-based surveys compared to mail, phone, and in-person surveys. [33], [34], [35], [36] In addition, several large-scale studies comparing online to mail survey modes have found that online surveys have lower overall response rates but yield higher item response rates on both open- and close-ended questions, suggesting higher data quality. [37], [38], [39], [40].

We report findings from a theoretically grounded online randomized controlled trial (RCT), evaluating the use of HIV prevention videos and a prevention website to deliver risk reduction content to HIV-negative, HIV-positive and untested MSM. The two HIV prevention videos used in this trial were designed to reflect issues specific to MSM, while incorporating social learning theory, situated cognition, and developmental learning theory into the dialogue, storyline, and realism of the characters [41], [42]; principles of these theories are reflected in the videos' use of realistic stories, recognizable character types, and conflict to promote critical thinking. [43] For this online trial, we hypothesized that between baseline and 60-day follow-up, there would be increases in HIV disclosure and HIV testing, and decreases in UAI among those randomized to an HIV prevention video or a prevention webpage, compared to those in the control condition.

## Methods

The protocol for this trial and supporting CONSORT checklist are available as supporting information; see Checklist S1 and Protocol S1. Survey outcome rates and methodological terminology are based on the reporting standards of the American Association for Public Opinion Research (AAPOR). [44].

### Study Design

This online 5-arm RCT, with a 1∶1 allocation ratio, compared the impact of three HIV prevention video conditions, an HIV prevention webpage, and a no-content control on three primary outcomes (HIV testing, HIV disclosure and UAI) at 60-day follow-up. The study design was based on an online pre-post test video-based intervention pilot conducted in 2005. [28] Since the retention rate for the pilot study was 54% with a 90-day follow-up period, investigators in the current study opted for a shorter, 60-day follow-up period to potentially increase the overall retention rate.

### Objectives

The primary objective of this 5-arm RCT was to assess the feasibility and efficacy of implementing a large-scale single-session online intervention, using HIV prevention videos or an HIV prevention webpage versus a no-content control, among sexually active U.S. MSM, aged 18 and over, who were recruited from four gay-oriented sexual networking websites.

### Participants

Eligibility criteria for the intervention were programmed into the online baseline survey. Participants had to: 1) identify as a man; 2) be age 18 or older; 3) reside in the U.S.; 4) provide an email address; 5) report oral or anal sex with a current male partner (new or not), and oral, anal, or vaginal sex with at least one new partner (male or female) in the previous 60 days; 6) and have the ability to read and respond in English. Men who completed the baseline survey but were ineligible for the intervention were automatically transferred to the exit page that contained links to health-related websites and hotlines.

### Sampling Frame

In April 2008, a banner ad was placed on four gay-oriented sexual networking websites for U.S. men. The demographic characteristics of the banner ad sampling frame are undefined as we do not know who was exposed to ad views. After several weeks of slow recruitment, one of the websites agreed to send emails through its internal system to all of its U.S. members (i.e., a list-based sampling frame of emails). Members of this site are automatically assigned an email address upon becoming a member, thus ensuring a valid user email address. The email sent to members contained a study banner, and all email recipients were considered potentially eligible for study inclusion. [44] Participants were recruited online between April and June 2008. A total of 609,960 emails were sent nationwide, with a 99.6% absorption rate (i.e., successful delivery of emails, indicating a high-quality sampling frame). The absorption rate was calculated by dividing the number of delivered emails (607,777) by all email invitations sent. [45] No incentives were offered to study participants.

### Ethics Statement

The institutional review boards at Public Health Solutions (a nonprofit organization in New York City) and the Centers for Disease Control and Prevention (CDC) approved all study procedures. A waiver of documentation of written consent was obtained, given the internet-based research approach. Men who clicked on the study banner ad provided informed consent online by reading the consent form and clicking agreement to participate in the baseline survey. Following completion of the baseline survey, participants who met the inclusion criteria for the RCT were provided a second consent form inviting them to participate in additional study activities.

### Interventions

The five study conditions included a: 1) dramatic video; 2) documentary video; 3) both dramatic and documentary videos; 4) prevention webpage; and 5) control (i.e., received no intervention content). Both the dramatic and documentary HIV prevention videos (http://hivbigdeal.org/) were designed to promote critical thinking about HIV disclosure, HIV testing, and condom use. The videos were based on social learning theories and strategies [41], [42], [46] that informed the instructional design and delivery of the online intervention along three important design dimensions: (1) the medium selected (i.e., video); [47], [48], [49], [50] (2) the degree of realism in the content; [51], [52] and (3) the finer-grained structure of the content, such as conflict between the characters to promote critical thinking. [41], [42], [53], [54], [55], [56].

Both HIV prevention videos were designed to tell the same story, through drama, on the one hand, and through documentary, on the other. Both videos provided positive and negative modeling examples of HIV disclosure to sex partners, which emphasized critical thinking and decision-making. Goals of the modeling examples conveyed by the prevention videos included increasing HIV disclosure awareness, serving as a refresher for risk reduction, and increasing a sense of responsibility to protect one’s sexual partners. [57], [58] *The Morning After* is a 9-minute dramatic video addressing sexual risk reduction and features 3 gay male friends, one of whom thinks he had unprotected sex with an HIV-positive man while intoxicated and seeks advice from friends. *Talking About HIV* is a 5-minute documentary video addressing sexual risk reduction through testimonials of HIV-positive men and was created with footage from the feature-length documentary, “Meth.” [59].

Participants randomized to both videos were provided the videos in random order. Men randomized to the prevention webpage were provided a CDC webpage that featured information about HIV among MSM, with links to prevention information and resources. Participants assigned to the control condition were only provided with links to HIV prevention resources following completion of the behavioral survey. All participants could view their assigned online content, though once they closed their web browser they could not view the content again.

### Follow-up

Participants were emailed 60 days post-baseline to complete the follow-up survey, which paralleled the baseline survey. A hyperlink was embedded in the email, and when clicked on, automatically transferred the participant to a second consent and follow-up survey. For those who did not respond to the follow-up email, we waited one week before sending a first reminder email, another week before sending the second reminder, and a third week before sending the final reminder (for an additional 21–30 days). The 60-day follow-up period was slightly extended to September 2008 (after receiving IRB approval) in order to send an additional email reminder to all intervention participants who did not complete the 60-day follow-up survey; this resulted in an additional 194 responses (12%).

### Outcomes

We hypothesized that between baseline and 60-day follow-up, there would be increases in HIV disclosure and HIV testing, and decreases in UAI among those randomized to an HIV prevention video or a prevention webpage, compared to those in the control condition. The three primary outcome measures were increased HIV testing, HIV disclosure, and decreased UAI with sex partners at 60-day follow-up. At baseline, HIV testing variables included ever testing for HIV and month/year of last test and the result. At follow-up, HIV testing was measured as receiving an HIV test during the 60-day follow-up period. HIV disclosure was defined as partial (i.e., asking or telling) or full (i.e., both asking and telling) with a partner in a sexual encounter. Anal intercourse was defined as ‘any’ insertive and/or receptive sex (yes/no). UAI was defined as ‘any’ unprotected (without a condom) insertive and/or receptive sex (yes/no). Men were asked about the three most recent sexual partners in the past 60 days beginning with the most recent. Due to a software programming error, baseline HIV disclosure data captured information for only the last partner reported in the past 60 days. To address this limitation, we compared primary outcome data between the last partner reported at baseline and the corresponding last partner reported during follow-up.

### Sample Size

Based on the prevalence of behaviors in our previous studies, we calculated true proportions and sample sizes using chi-square tests for this 5-group design. We estimated that by enrolling approximately 600 men per group and retaining 70% at 60 days follow-up, we would have 80% power at a 5% alpha level to detect behavioral change differences between 10–15% for the dichotomous primary outcomes (increased HIV disclosure, testing, and decreased UAI) between the video conditions and control condition at 60-day follow-up. As an example of the actual range of power that we had to detect a difference between the control and video conditions, the power ranged from 57% to 82% across the individual video conditions for asking a sex partner’s HIV status at follow-up. After combining the video conditions, we had 90% power to detect a difference in HIV disclosure at follow-up.

### Randomization

Those who consented were randomized into one of the five study conditions using a computerized randomization program, which included a blocking scheme to balance randomization across the study conditions using a non-deterministic algorithm. [60], [61] The decisecond of the participant’s “click” determined study condition assignment, which continued throughout the balancing process, resulting in a study sample that was balanced within a 1% range.

### Statistical Methods

Once participants consented and were randomized, they were kept in their original assignment condition and sent a link to the 60-day follow-up survey (i.e., intention to treat [ITT]). Thus, we used an ITT approach for primary outcome analyses. Since randomization was not stratified, for Tables 1 and 2, we took a conservative approach and tested for baseline differences across conditions. Chi-square tests assessed group-level comparisons of the dichotomous primary outcome measures at 60 days post-intervention. Follow-up sample characteristics were assessed using bivariate, rather than multivariate, logistic regression, as several characteristics were highly correlated. Due to reduced power from low retention (53%), the 3 video conditions were pooled for all outcome analyses. Statistical analyses were conducted using SPSS 20 for Windows. [62].

**Table 1:** Baseline Demographic and Behavioral Characteristics by Randomization Group.

Characteristic	Total(N = 3,092)	PooledVideosn = 1,874	No-ContentControln = 609	PreventionWebpagen = 609	Pooled Videovs. Control	PreventionWebpage vs.Control
	n (%)	n (%)	n (%)	n (%)	p-value	p-value
**Age**, n = 3,092						
18–24	441 (15)	262 (14)	79 (13)	100 (16)		
25–29	379 (12)	210 (11)	86 (14)	83 (14)		
30–39	741 (24)	454 (24)	144 (24)	143 (23)		
40–49	965 (31)	599 (32)	184 (30)	182 (30)		
50+	566 (18)	349 (19)	116 (19)	101 (17)	.38	.47
**Race/Ethnicity**, n = 3,084						
White	2,503 (81)	1,540 (82)	488 (80)	475 (78)		
Black	126 (4)	69 (4)	32 (5)	25 (4)		
Hispanic	275 (9)	169 (9)	52 (9)	54 (9)		
Asian/Mixed/Other Race	180 (6)	89 (5)	37 (6)	54 (9)	.19	.24
**Education**, n = 3,089						
High School or less	322 (10)	185 (10)	67 (11)	70 (11)		
Some college or enrolled	1,076 (35)	649 (35)	222 (37)	205 (34)		
College degree or more	1,691 (55)	1,037 (55)	320 (52)	334 (55)	.44	.59
**Income**, n = 2,909						
Less than $50,000	1,480 (51)	892 (51)	301 (52)	287 (50)		
$50,000 or more	1,429 (49)	866 (49)	278 (48)	285 (50)	.60	.54
**HIV Status**, n = 3,079						
HIV-Negative	2,323 (75)	1,420 (76)	459 (76)	444 (73)		
HIV-Positive	532 (17)	316 (17)	101 (17)	115 (19)		
Untested	224 (8)	134 (7)	43 (7)	47 (8)	.99	.52
**Male Anal Sex Partners**						
Lifetime, n = 3,034						
1–10	755 (25)	446 (24)	157 (26)	152 (26)		
11–50	911 (30)	554 (30)	170 (29)	187 (31)		
51–100	451 (15)	263 (14)	98 (16)	90 (15)		
101–500	507 (17)	320 (18)	95 (16)	92 (15)		
501+	410 (13)	257 (14)	78 (13)	75 (13)	.49	.86
Past Year, n = 3,082						
0	158 (5)	94 (5)	31 (5)	33 (6)		
1–5	1,357 (44)	804 (43)	265 (44)	288 (47)		
6–10	630 (20)	390 (21)	126 (21)	114 (19)		
11–50	664 (22)	413 (22)	129 (21)	122 (20)		
51+	273 (9)	167 (9)	57 (9)	49 (8)	.99	.66

Overall sample includes participants who reported male partners only (n = 2,950, 95%), male and female partners (n = 113, 4%), and male and transgender partners (n = 16, 1%); 13 participants did not report one-on-one sexual encounters and did not have encounter-specific data.

**Table 2:** Sexual Behavior in the 60 Days Prior to Enrollment by Randomization Group.

60 Days Prior to Baseline	Total(n = 3,092)	PooledVideosn = 1,866	No-ContentControln = 606	PreventionWebpagen = 607	PooledVideo vs.Control	PreventionWebpage vs.Control
	n (%)	n (%)	n (%)	n (%)	p-value	p-value
**HIV disclosure with Last Partner** * n = 3,079						
Asked (y, n)						
Last partner (main or non-main)	1,352 (44)	809 (43)	261 (43)	282 (47)	.90	.24
Non-main	1,175 (38)	700 (40)	219 (39)	256 (44)	.67	.07
Main	177 (6)	109 (9)	42 (11)	26 (7)	.39	.11
Told (y,n)						
Last partner (main or non-main)	1,621 (53)	973 (52)	313 (52)	335 (55)	.83	.22
Non-main	1,401 (46)	847 (49)	263 (47)	291 (52)	.57	.14
Main	220 (7)	126 (12)	50 (15)	44 (14)	.29	.81
Asked and Told (y,n)						
Last partner (main or non-main)	1,013 (33)	609 (33)	187 (31)	217 (36)	.42	.07
Non-main	879 (29)	529 (30)	154 (27)	196 (33)	.21	**.02**
Main	134 (4)	80 (6)	33 (7)	21 (5)	.32	.18
**Tested for HIV, past 60 days** † n = 2,495						
Yes	445 (18)	266 (17)	88 (18)	91 (19)	.75	.69
**Any Anal Intercourse** ‡ n = 3,079						
Yes	2,460 (80)	1,503 (81)	469 (77)	488 (80)	.09	.20
No	619 (20)	363 (19)	137 (23)	119 (20)		
**Any Unprotected Anal intercourse** ‡ n = 3,079						
Yes	1,778 (58)	1,065 (57)	344 (57)	369 (61)	.89	.15
No	1,301 (42)	801 (43)	262 (43)	238 (39)		
Among men with any non-main partners, n = 2,896						
Yes	1,673 (58)	1,000 (57)	323 (57)	350 (61)	.96	.13
No	1,223 (42)	759 (43)	244 (43)	220 (39)		
Among men with only main partners, n = 183						
Yes	105 (57)	65 (61)	21 (54)	19 (51)	.45	.83
No	78 (43)	42 (39)	18 (46)	18 (49)		
**STI diagnosis** §						
Yes	457 (15)	270 (15)	97 (16)	90 (15)	.37	.57
**Median partners**						
(Male) oral sex partners only	2	3	2	3	.23	.51
(Male) anal sex partners	2	2	2	2	.53	.25

Some variables have missing data.

* Baseline HIV disclosure data were only available for the last encounter in the 60 days prior to baseline. In addition, 13 cases did not report any past 60-day one-on-one sexual encounters and therefore had no data for these questions.

† HIV testing among HIV-negative and untested status men.

‡ Combined male and female partner data for up to the last 3 sexual encounters; 51 cases included female partners.

§ STI  =  sexually transmitted infections. Both the baseline and follow-up behavioral surveys inquired about any STIs diagnosed by a nurse or physician in the past 60 days, which included chancroid, chlamydia, gonorrhea, herpes, human papillomavirus, lymphogranuloma venereum, nongonococcal urethritis, syphilis, and hepatitis A, B, or C. Significant findings are in bold.

**Figure 1:**
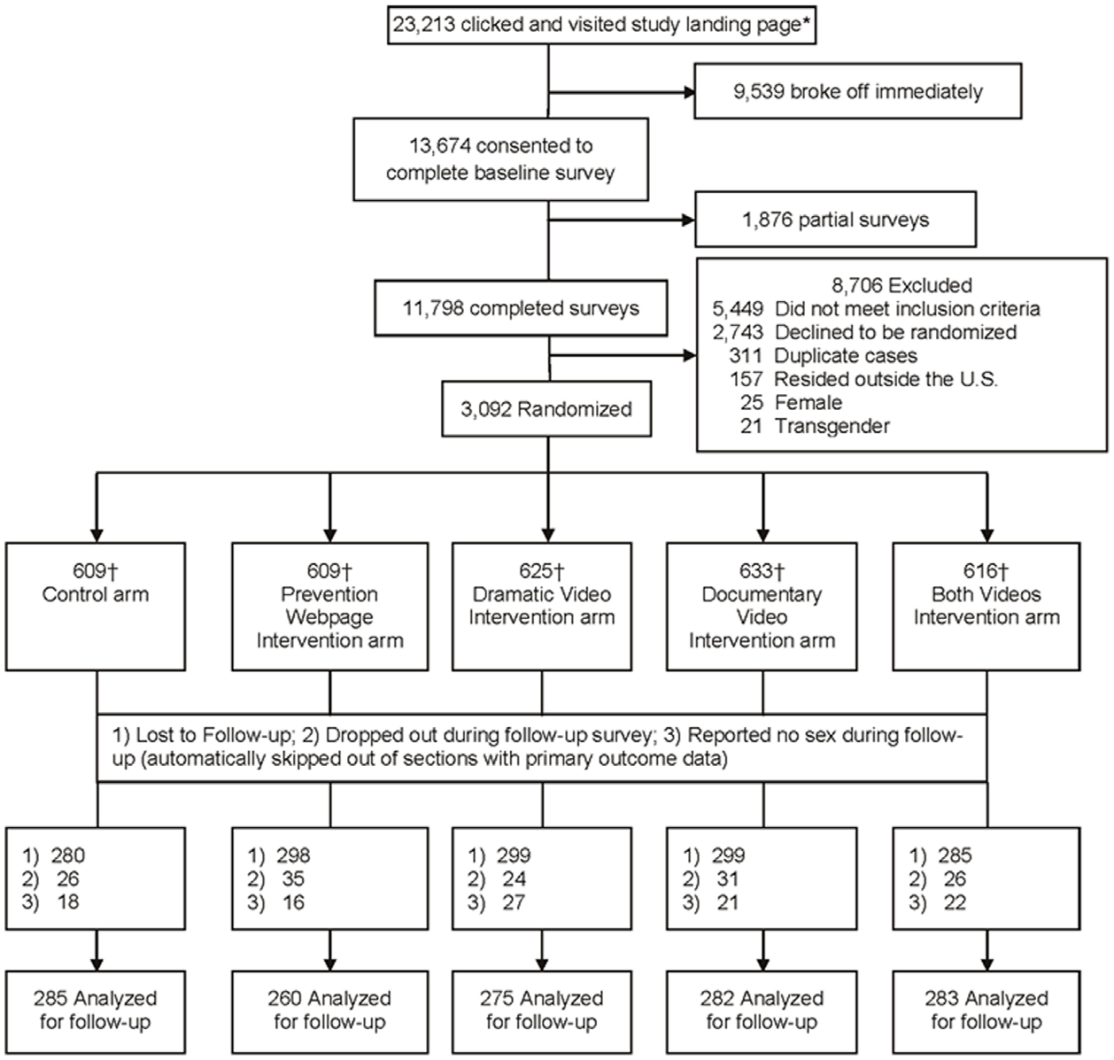
Study flow chart. *Recruited via email (n = 609,960) or banner ad (the number of impressions that men were exposed to are not available). †Completed baseline behavioral survey.

Analyses in Tables 3 and 4 account for losses and exclusions, which include being lost to follow-up (n = 1,461), dropping out during the follow-up survey (n = 142), and reporting no sexual activity during follow-up (and thus being automatically skipped out of the sexual encounter sections, but not the HIV testing section) (n = 104) (Figure 1). In Table 3, for group-level comparisons of primary outcomes at 60-day follow-up, the control condition was compared to the pooled video condition and prevention webpage condition separately. In Table 4, within-person changes for primary outcomes from baseline to 60-day follow-up were compared using McNemar’s test for paired data in the pooled video, webpage, and control conditions. Finally, we conducted sensitivity analysis and assessed follow-up non-response bias to examine the impact of attrition.

**Table 3:** Primary Outcome Behaviors at 60-Day Follow-up.

Outcome	Pooled Videos	No-ContentControl	PreventionWebpage	Pooled Videovs. Control	PreventionWebpage vs.Control
	n (%)	n (%)	n (%)	OR (95% CI)	OR (95% CI)
**Among all men**, n = 1,385					
*HIV disclosure with sex partners* *	n = 840	n = 285	n = 260		
Asked	467 (56)	129 (45)	130 (50)	**1.51 (1.16–1.98)**	1.21 (0.86–1.69)
Told	569 (68)	196 (69)	181 (70)	0.95 (0.71–1.27)	1.04 (0.72–1.49)
Asked and Told	391 (47)	113 (40)	116 (45)	**1.32 (1.01–1.74)**	1.23 (0.87–1.72)
*Any UAI* †, *n = 1,603*	479/975 (49)	157/322 (49)	157/306 (51)	1.02 (0.79–1.31)	1.11 (0.81–1.51)
**Among men with any non-main partners**, n = 1,282					
*HIV disclosure with sex partners* *	n = 774	n = 265	n = 243		
Asked	433 (56)	122 (46)	120 (50)	**1.49 (1.13–1.97)**	1.15 (0.81–1.64)
Told	524 (68)	183 (69)	170 (70)	0.94 (0.69–1.27)	1.06 (0.72–1.55)
Asked and Told	362 (47)	107 (40)	107 (44)	1.29 (0.98–1.72)	1.17 (0.82–1.67)
*Any UAI* †, *n = 1,273*	442/768 (58)	145/262 (55)	145/243 (60)	1.09 (0.83–1.45)	1.19 (0.84–1.70)
**Among men who only had main partners**, n = 104					
*HIV disclosure with sex partners* *	n = 66	n = 20	n = 18		
Asked	34 (52)	7 (35)	10 (57)	1.97 (0.69–5.57)	2.32 (0.63–8.58)
Told	45 (66)	13 (65)	11 (61)	1.15 (0.40–3.31)	0.85 (0.23–3.17)
Asked and Told	29 (44)	6 (30)	9 (50)	1.83 (0.63–5.35)	2.33 (0.62–8.82)
*Any UAI* †, *n = 101*	37/65 (57)	12/19 (63)	12/17 (71)	0.77 (0.27–2.21)	1.40 (0.35–5.67)
**HIV Testing since baseline**, n = 1,116‡ 142 (21)		48 (20)	41 (20)	1.08 (0.75–1.55)	0.97 (0.61–1.54)
**STI diagnosis since baseline**, n = 1,375§ 47 (6)		16 (6)	13 (5)	0.99 (0.56–1.79)	0.84 (0.39–1.79)
**Male Sex Partners** (past 60 days)					
median anal sex	2	2	2	0.32	0.54
median oral sex	3	3	3	0.88	0.92

All variables have missing data.

* HIV disclosure and anal sex variables include sex partner data for up to the 3 last sexual encounters; 1,720 did not report disclosure data due to loss to follow-up (1,461) or drop-out during the follow-up survey (142), no sex during follow-up (104), or only multiple-partner encounter data at baseline and thus no one-on-one encounter data (13).

† Unprotected anal intercourse (UAI) was defined as ‘any’ unprotected (without a condom) insertive and/or receptive sex (yes/no).

‡ HIV Testing among HIV-negative and previously untested status men.

§ Since baseline, 74 men (5%) reported bacterial and/or newly diagnosed viral sexually transmitted infections (STIs), which included chancroid, chlamydia, gonorrhea, herpes, human papillomavirus, lymphogranuloma venereum, nongonococcal urethritis, syphilis, and hepatitis A, B, or C. Significant findings are in bold.

**Table 4:** McNemar’s Post Hoc Tests for Primary Outcomes: Baseline to 60-Day Follow-up Behaviors.

*60 days prior to Baseline* *to 60 days prior* *to Follow-up*	TotalN	Pooled VideosBehaviorChangeS1/S2 *		OR (95% CI)	TotalN	WebpageBehaviorChangeS1/S2*		OR (95% CI)	TotalN	No-ContentControlBehaviorChangeS1/S2*		OR (95% CI)
		No toYes	Yes toNo			No toYes	Yes toNo			No toYes	Yes toNo	
		N	n			n	n			N	n	
**HIV Disclosure with Least Recent Partner**												
Asked partner’s HIV status												
Overall	831	115	133	0.86 (0.66–1.12)	257	28	46	**0.61 (0.37–0.99)**	280	33	55	**0.60 (0.38–0.94)**
HIV-negative participants	633	96	104	0.92 (0.69–1.23)	186	21	34	0.62 (0.34–1.10)	220	27	44	0.61 (0.37–1.01)
HIV-positive participants	159	13	24	0.54 (0.25–1.11)	56	6	10	0.60 (0.18–1.82)	44	5	9	0.56 (0.15–1.85)
Told partner HIV status												
Overall	831	127	151	0.84 (0.66–1.07)	257	46	39	1.18 (0.75–1.86)	280	54	51	1.06 (0.71–1.58)
HIV-negative participants	633	100	118	0.85 (0.64–1.12)	186	38	29	1.31 (0.79–2.20)	220	46	38	1.21 (0.77–1.91)
HIV-positive participants	159	23	29	0.79 (0.44–1.42)	56	7	6	1.17 (0.34–4.20)	44	5	9	0.56 (0.15–1.85)
Asked and told HIV status												
Overall	831	104	136	**0.76 (0.59–0.99)**	257	35	41	0.85 (0.53–1.37)	280	41	44	0.93 (0.59–1.46)
HIV-negative participants	633	92	107	0.86 (0.64–1.15)	186	28	28	1.00 (0.57–1.75)	220	37	37	1.00 (0.62–1.62)
HIV-positive participants	159	9	26	**0.35 (0.14–0.76)**	56	6	10	0.60 (0.18–1.82)	44	4	6	0.67 (0.14–2.81)
**Self-reported HIV Testing**												
HIV-negative/untested men	595	104	78	1.33 (0.99–1.81)	182	28	20	1.40 (0.76–2.62)	211	27	20	1.35 (0.73–2.54)
**Reported any UAI** ‡												
Overall	991	121	198	**0.61 (0.48–0.77)**	251	30	71	**0.42 (0.27–0.66)**	329	43	61	0.70 (0.47–1.06)
Any non-main partners	758	109	109	1.00 (0.76–1.32)	226	25	40	0.63 (0.36–1.06)	257	35	33	1.06 (0.64–1.76)
Only main partners	14	0	2	0.00 (0.00–5.33)	5	1	0	0.00 (0.01–39.00)	5	0	1	0.00 (0.00–39.00)
HIV-negative participants	743	98	140	**0.70 (0.54–0.91)**	182	22	51	**0.43 (0.25–0.72)**	257	38	44	0.86 (0.54–1.36)
HIV-positive participants	194	17	45	**0.38 (0.20–0.67)**	53	6	12	0.50 (0.15–1.44)	52	4	13	0.31 (0.07–1.00)
**Serodiscordant UAI with any Non-Main partners**												
HIV-negative participants	580	66	73	0.90 (0.64–1.28)	162	22	20	1.10 (0.57–2.13)	200	22	20	1.10 (0.57–2.13)
HIV-positive participants	125	18	34	**0.53 (0.28–0.96)**	42	3	11	0.27 (0.05–1.03)	32	4	5	0.80 (0.16–3.72)

* Proportions in rows may not add to 1.00 due to rounding;

† All baseline (Survey 1) and follow-up (Survey 2) chi-square and p-values are paired data, exact McNemar tests;

‡ Unprotected anal intercourse. The McNemar's odds ratio was calculated by dividing the proportion reporting ‘no to yes’ (from baseline to follow-up) in the numerator over the proportion reporting ‘yes to no’ (from baseline to follow-up) in the denominator. *p≤.05, **p≤.01, ***p≤.001.

## Results

### Participant Flow

One of the four sexual networking websites agreed to send emails to all of its U.S. members (n = 609,960). A total of 23,213 (3.8%) men clicked on the study recruitment email hyperlink or banner ad that took them to the study landing page (Figure 1). Of those, 9,539 broke off from the landing page immediately, and 13,674 consented to participate in the baseline survey. Among men who consented, 11,798 (86%) completed the baseline survey and 1,876 (14%) had partial baseline surveys. Overall, the email recruitment response rate (AAPOR RR1) was 1.9% for completed cases and (AAPOR RR2) 2.2% with partials included. The number of banner ad impressions men were exposed to was not available from the websites, therefore we could not calculate a click-through-rate for men recruited through banner ads.

Among the consenting 11,798, the following were excluded: 157 residing outside of the US; 25 female, 17 female-to-male transgender, and 4 male-to-female transgender; 311 duplicate cases were detected and excluded. In addition, 5,449 men were ineligible for study enrollment, and 2,743 men were eligible but refused participation. A total of 3,092 participants were eligible and randomized into the study as follows: 609 in the control arm, 609 in the prevention webpage arm, 625 in the dramatic video arm, 633 in the documentary video arm, and 616 in the dramatic/documentary video arm (Figure 1). The drop-out rate for each study arm is provided in Figure 1.

### Baseline Characteristics

Tables 1 and 2 describe the demographic and behavioral characteristics of the 3,092 randomized participants at baseline. Overall, respondents were predominantly white with high income and education, and were from every U.S. state. The median age was 39 (range 18 to 81). In the last sexual encounter within the past 60 days, 44% asked their partner’s HIV status, 53% told a partner their HIV status, and approximately one-third reported full disclosure (both asking and telling). No differences were found across study conditions at baseline for the primary outcome behaviors, except for a higher prevalence of asking/telling among men in the webpage condition versus the control. This was considered due to chance as participants were unaware of study hypotheses. Regarding past 60-day sexual behavior with partners in up to the last 3 sexual encounters, 80% reported anal intercourse and 58% reported UAI. Overall, 17% self-reported being HIV-positive. Among HIV-negative men, 18% reported getting an HIV test within the 60 days prior to baseline and 69% were tested in the past year (data not shown). In the past 60 days, most participants reported at least 3 sexual partners (72%), followed by 2 (17%), and 1 (10%); 13 men (1%) reported only multiple-partner encounters and therefore did not answer this section.

#### Self-Selection characteristics

Among randomized respondents, 32% (n = 976) were recruited through banner ads and 68% (n = 2,116) through an e-mail member list. Most (93%) respondents recruited via banner ads were from the same sexual networking site that sent the study e-mail. Compared to men recruited through emails, men recruited via banner ads were significantly more likely to complete 60-day follow-up (58% vs. 50%, OR 1.39, p<.001). No demographic or primary outcome differences were found between the two recruitment methods, except for HIV status; HIV-positive men were significantly more likely to have been recruited through a banner ad than by email (20% vs. 16%, OR 1.31, p<.01) (data not shown).

### Follow-up Retention Sample Characteristics

Compared to men who did not complete 60-day follow-up, men who did complete follow-up were significantly older (t = 7.27, p<.001), and more likely to be white (versus black OR 0.59, p<.01; Hispanic OR 0.71, p<.01; mixed/other race OR 0.60, p = .001), college-educated (OR 1.67, p<.001), HIV-positive (versus negative or untested, OR 1.35, p<.01), and earn more than $50,000 per year (OR 1.33, p<.001). Men who completed follow-up were also significantly more likely to have non-main partners at baseline (OR 1.59, p<.01) and to report more than 100 lifetime anal sex partners (OR 1.34, p<.01) than men who did not complete follow-up.

### Outcomes

Overall retention at 60-day follow-up was 53% (n = 1,631) and there was no differential attrition by study condition or baseline demographic and behavioral characteristics in Tables 1 and 2.

Figure 1 lists the reasons and rates of drop-out per condition. The median duration between completion of baseline intervention activities and the 60-day post-baseline assessment was 61 days.

#### Primary differences across treatment conditions at follow-up

At follow-up (Table 3), men in the pooled video condition were significantly more likely than men in the control condition to report both partial HIV serostatus disclosure (“asking”, OR 1.51) and full disclosure (“asking and telling”, OR 1.32) in their sexual encounters in the past 60 days. Among men with non-main partners, those in the pooled video condition were significantly more likely than men in the control condition to ask (OR 1.49); between the two conditions, asking and telling approached significance (OR 1.29). No disclosure differences were seen for men with main partners. Among HIV-negative and untested men who completed follow-up (n = 1,116), 21% reported getting an HIV test; however there were no differences across study conditions. Eight men who self-reported never testing at baseline were tested within the 60-day follow-up period and reported a negative test result. No difference in sexual behavior was observed between conditions, with 68% reporting anal sex (data not shown) and 49% reporting UAI during follow-up. No differences were found between the prevention webpage and control condition for the primary outcomes.

#### Behavior change from baseline to follow-up

With McNemar’s test (Table 4), we examined within-person behavior changes using paired data from baseline to 60-day follow-up by study condition (pooled videos, webpage, and control) for the primary outcomes: HIV disclosure, UAI, and HIV testing. Significant decreases in UAI were seen from baseline to follow-up within the digital media conditions (OR 0.61, OR 0.42). HIV-negative men in both the pooled video and webpage conditions reported significant reductions in UAI from baseline to follow-up (OR 0.70, OR 0.43), while HIV-positive participants only in the pooled video condition reported a significant decrease in UAI (OR 0.38). However, HIV-positive men in the pooled video condition also reported a significant reduction in UAI with HIV-negative and unknown status non-main partners (OR 0.53) from baseline to follow-up.

Contrary to expectations, men in both the webpage and control conditions had significant reductions in asking their partner’s HIV status at follow-up compared to baseline (OR 0.61, OR 0.60). For full disclosure, men in the pooled video condition were significantly less likely to ask and tell, overall (OR 0.76), and among HIV-positive participants (OR 0.35), at follow-up compared to baseline. Regarding HIV testing from baseline to follow-up, no changes were seen in any of the conditions.

#### Sensitivity analysis

Comparing men who did and did not complete follow-up, we assessed the potential impact of attrition by conducting sensitivity analyses. We assessed HIV disclosure at follow-up with the last sexual partner for parsimony and made two different assumptions about HIV disclosure among those lost to follow-up. First, we assumed that all men lost to follow-up were disclosers and found that asking a sex partner’s HIV status would be significantly higher in the pooled video condition than in the control (79% vs. 74%, OR 1.38, 95% CI 1.10–1.72). Second, we assumed that all those lost to follow-up were non-disclosers and found that there would also be a significant difference, albeit smaller, in asking between conditions (26% vs. 22%, OR 1.25, 95% CI 1.00–1.57).

#### Follow-up non-response bias

To assess whether men who did not complete follow-up differed significantly on the primary outcome estimates from those who did complete follow-up, we tested for non-response bias. The full sample baseline primary outcome estimates for past 60-day behaviors were 44% for asking HIV status, 53% for telling HIV status, 58% for any UAI, and 18% for HIV testing. Using the baseline primary outcome estimates, we calculated follow-up non-response bias by subtracting the mean difference between men who responded to follow-up and the baseline sample, divided by the baseline sample estimate. Men who did complete follow-up had lower baseline mean estimates than men who did not complete follow-up for the baseline primary outcomes asking HIV status (41% vs. 47%), telling HIV status (52% vs. 53%), reporting any UAI (57% vs. 58%), and HIV testing (16% vs. 17%). We found significant non-response bias for asking HIV status, though the magnitude was small and was biased towards the null. Thus, men who completed the follow-up survey were significantly less likely to have asked their last sexual partner’s HIV status than men who did not complete the follow-up survey (41% vs. 47%, p = .001). No differences were found between men who did and did not complete follow-up for the other primary outcomes.

## Discussion

Findings from this online RCT of MSM recruited from sexual networking websites suggest that a low cost, brief digital media intervention conducted completely online can increase HIV disclosure to sexual partners and decrease sexual risk. Theoretically grounded HIV prevention videos, designed to engage critical thinking, and an HIV prevention website were evaluated for their impact on HIV disclosure, UAI, and HIV testing. MSM participating in the online trial were predominantly white with high income and education and were from every U.S. state. As reporting a new sex partner at baseline was one eligibility criterion for inclusion into the online trial, men participating in our study reported considerable HIV transmission risk at enrollment. On average, men reported 2 anal sex partners in the past 60 days, with more than half reporting UAI with non-main partners. Men who completed 60-day follow-up differed from men who did not complete follow-up by demographic and behavioral characteristics; most notably, men completing follow-up were significantly more likely to be HIV-positive, report non-main partners at baseline, and report more lifetime anal sex partners than men not completing follow-up.

### Group-Level Effects

At 60-day follow-up, modest group-level effects were seen for HIV disclosure. Men in the pooled video condition were significantly more likely than men in the control condition to report full (“asking and telling”) and partial (“asking”) HIV disclosure with sexual partners. Among men who had non-main partners during follow-up, partial HIV disclosure was significantly higher in the video condition than control condition. Significant changes in sexual behavior and HIV testing were not seen at the group level.

### Within-Person Behavior Change

Contrary to expectations, some men were significantly less likely to disclose their HIV status to sex partners from baseline to 60-day follow-up. In contrast, within-person effects were found for reduced UAI at follow-up compared to baseline, with men reporting significant reductions in UAI in both the pooled video (OR 0.61) and webpage conditions (OR 0.42). The reduction in UAI among men in the pooled video condition is similar to a previously published one-group, pre-post pilot intervention – using one of the same videos used in the current trial – among MSM recruited from one of the same gay-oriented sexual networking websites that assessed within-person reduction of UAI (OR 0.55). [28] This finding suggests that watching a video about two gay men negotiating HIV disclosure and sexual risk can promote critical thinking and lead to reduced sexual risk.

From baseline to 60-day follow-up, HIV-negative men reported significant reductions in UAI in both the pooled video and webpage condition. HIV-positive men in the pooled video condition also reported significant reductions in UAI, but the most striking finding for HIV-positive men in the video condition was their significant reduction in UAI with HIV-negative or unknown status partners at follow-up compared to baseline (OR 0.53). It appears that that this low intensity digital media intervention may have resonated most with sexually active HIV-positive MSM, who may not be reached by traditional offline prevention messages.

While HIV disclosure during follow-up was significantly higher across several sex partners (i.e., video versus control condition) it was significantly lower when examined by participants’ baseline-to-follow-up corresponding partner. UAI was also significantly lower with corresponding partners from baseline to follow-up, which suggests that, rather than having a potentially awkward discussion about sex and serostatus, some men may have opted not to have unprotected sex. Disclosure to sexual partners and its relationship to sexual risk is complex and varies by partner type and venue [63], [64], [65] but is a critical component of safer sexual behavior, particularly among those with HIV. [66].

### Limitations

Historically, online research has had lower retention rates than offline research as there are fewer social constraints compared to in-person interviewing. [32] In the current study that was conducted in 2008, online retention at 60 days post-intervention was 53%, which is low but comparable to other online prevention interventions that were also conducted several years ago. [28], [67], [68] Another factor potentially impacting retention in this study was the lack of incentives, which likely contributed to lower retention rates. [69] Online delivery of the study provided an efficient means to reach a large sample size relatively quickly, but given the size of the sample, the study was unable to provide incentives for participation. Offline studies have also found pronounced differences in retention rates among those offering and not offering incentives. [70], [71].

Within a given study, non-response bias can occur when respondents differ from non-respondents, though non-response, in and of itself, does not mean that the data are inherently biased. [72] Observed differences between responders and non-responders do not necessarily indicate response bias, unless the differences are related to the study outcomes. [73] Across the primary outcomes, only one variable, asking a sex partner’s HIV status, was found to be consistently higher at baseline among men who did not complete follow-up than men who did. In sensitivity analysis, men in the video condition reported significantly higher asking of their partner’s HIV status than men in the control condition, demonstrating a robust finding. In follow-up non-response bias analyses we found a small but significant difference in asking a sex partner’s HIV status, with non-responders reporting higher disclosure, indicating that non-response bias was present but biased towards the null. Thus, men who completed the online follow-up survey were less likely to disclose at baseline and may have had a greater need for an online risk reduction intervention. These findings may not be generalizable to all men who access gay-oriented sexual networking websites or who may be exposed to study emails or banner ads.

### Conclusions

Even within the context of these limitations, in this predominantly white sample of MSM recruited from one of the largest U.S. sexual networking websites for gay men, there was a modest effect from this online digital media intervention, with an overall increase in HIV disclosure and reduced sexual risk in both HIV-positive and HIV-negative men. The reduction in serodiscordant UAI among HIV-positive participants suggests that the videos resonated most with HIV-positive men, who may have been more amenable to change in an online intervention setting, [74] as a relatively high proportion enrolled into the study, were significantly more likely to complete follow-up than HIV-negative and untested men, and significantly reduced sexual risk behavior.

The development of online HIV prevention interventions is a burgeoning field. But in the overall landscape of HIV prevention, how do technology-based interventions fit in? There has been a recent push in HIV prevention for “combination prevention.” [75] This multifaceted approach combines biomedical prevention tools and behavioral science, with the goal of targeting and engaging specific at-risk populations and communities to reduce HIV transmission risk behaviors with a sustained impact. With the recent FDA approvals of an over-the-counter in-home HIV oral rapid test, [76] and the use of pre-exposure prophylaxis (PrEP) to reduce the risk of sexually-acquired HIV, [77] Internet-based HIV prevention interventions including digital media are uniquely positioned to provide a critical platform to promote new HIV prevention technologies among a broad audience of at-risk MSM.

The use of theoretically grounded dramatic or documentary video designed to engage critical thinking, in combination with biomedical science, is a largely unexplored area. Brief, widely available, effective digital media interventions like the HIV prevention videos and webpage described here may serve as a complementary part of an overall strategy for increasing HIV disclosure and reducing sexual risk. Even if modestly effective, online social marketing campaigns have the potential to expose a much broader audience to brief, digital media prevention interventions. Delivering HIV prevention content to MSM online where they seek sexual partners is an efficient way to reach large samples of geographically dispersed at-risk men and has the potential for a public health impact through addressing the specific needs and circumstances of the target population, and by changing individual knowledge, motivations, and community norms.

## Supporting Information

Checklist S1 **CONSORT Checklist.**
(DOCX)

Protocol S1 **Trial Protocol.**
(DOC)
