# Supplementary material for: An Online Randomized Controlled Trial Evaluating HIV Prevention Digital Media Interventions for Men Who Have Sex with Men
Source: PLoS One. 2012 Oct 2;7(10):e46252. doi: 10.1371/journal.pone.0046252 (PMC3462792; doi:10.1371/journal.pone.0046252)
Supplement: Protocol S1 — Trial Protocol. (DOC) [file pone.0046252.s002.doc]

Novel Internet-based Interventions to Reduce Sexual Risk among

Men Who Have Sex with Men

**Research Protocol**

Cooperative Agreement Number: UR6 PS000415

CDC, NCHHSTP, Division of HIV/AIDS Prevention, Prevention Research Branch

Site: Public Health Solutions

Note: On 2/29/2008, MHRA will be changing its name to Public Health Solutions. The protocol reflects this change throughout.

Project Period: 09/01/2006 - 12/31/2008

**Principal Investigator: Sabina Hirshfield, PhD**

Senior Research Scientist

Public Health Solutions

220 Church Street, 5^th^ Floor

New York, NY 10013

T 646 619.6676

F 646 619.6777

**Table of Contents**

[**I. PROJECT OVERVIEW** 4](#__RefHeading___Toc190847520)

[**Protocol Summary** 4](#__RefHeading___Toc190847521)

[**Study Site** 5](#__RefHeading___Toc190847522)

[**Investigators/Collaborators** 5](#__RefHeading___Toc190847524)

[**II. INTRODUCTION** 5](#__RefHeading___Toc190847525)

[**Literature Review and Justification for the Study** 5](#__RefHeading___Toc190847526)

[**Intended/Potential Use of the Study Findings** 7](#__RefHeading___Toc190847528)

[**Specific Aims and Hypotheses** 7](#__RefHeading___Toc190847529)

[**III. PROCEDURES / METHODS** 8](#__RefHeading___Toc190847530)

[**Study Design and Location** 8](#__RefHeading___Toc190847531)

[**Procedures, Duration, Experimental Procedures** 8](#__RefHeading___Toc190847532)

[**Evaluation of Branches** 9](#__RefHeading___Toc190847533)

[**Theoretical Framework for Standard Text-Based Prevention Web Page** 9](#__RefHeading___Toc190847534)

[**Theoretical Framework for Video Interventions** 9](#__RefHeading___Toc190847535)

[**Audience/Stakeholder Participation** 12](#__RefHeading___Toc190847536)

[**Risks to Respondents** 12](#__RefHeading___Toc190847537)

[**Protection against Risks** 12](#__RefHeading___Toc190847538)

[**Potential Benefits** 14](#__RefHeading___Toc190847539)

[**Study Timeline** 14](#__RefHeading___Toc190847540)

[**IV. STUDY POPULATION** 17](#__RefHeading___Toc190847541)

[**General Description** 17](#__RefHeading___Toc190847542)

[**Intervention Participant Inclusion Criteria** 17](#__RefHeading___Toc190847543)

[**Intervention Participant Exclusion Criteria** 18](#__RefHeading___Toc190847544)

[**Exclusion of Women and Transgender Persons** 18](#__RefHeading___Toc190847545)

[**Exclusion of Children** 18](#__RefHeading___Toc190847546)

[**Estimated Number of Participants** 18](#__RefHeading___Toc190847547)

[**Sampling, Sample Size, and Statistical Power** 19](#__RefHeading___Toc190847548)

[**Participant Recruitment and Enrollment Procedures** 20](#__RefHeading___Toc190847549)

[**Randomization Procedures** 21](#__RefHeading___Toc190847550)

[**Attrition and Retention Efforts** 23](#__RefHeading___Toc190847551)

[**Incentives** 24](#__RefHeading___Toc190847552)

[**Informed Consent Procedures** 24](#__RefHeading___Toc190847553)

[**Justification to Waive Documentation of Consent** 25](#__RefHeading___Toc190847554)

[**Study Instruments and Variables** 25](#__RefHeading___Toc190847555)

[**Standardized Measures Included in Instruments** 26](#__RefHeading___Toc190847556)

[**Primary and Secondary Outcome Variables: Baseline & (60-day) Follow-up Period** 28](#__RefHeading___Toc190847557)

[**Additional Measurements** 29](#__RefHeading___Toc190847558)

[**Outcomes and Minimum Meaningful Differences** 29](#__RefHeading___Toc190847559)

[**Training for Study Personnel** 29](#__RefHeading___Toc190847560)

[**V. DATA HANDLING AND ANALYSIS** 29](#__RefHeading___Toc190847561)

[**Data Collection Procedures** 31](#__RefHeading___Toc190847562)

[**Protection of Privacy/Confidentiality** 31](#__RefHeading___Toc190847563)

[**Lack of Need for Assurance or Certificate of Confidentiality** 32](#__RefHeading___Toc190847564)

[**Information Management and Analysis Software** 32](#__RefHeading___Toc190847565)

[**Data Management** 32](#__RefHeading___Toc190847566)

[**Quality Assurance** 33](#__RefHeading___Toc190847567)

[**Bias in Data Collection, Measurement, and Analysis** 33](#__RefHeading___Toc190847568)

[**Intermediate Reviews and Analysis** 34](#__RefHeading___Toc190847569)

[**Response to New and Unexpected Findings/Changes in Environment** 34](#__RefHeading___Toc190847570)

[**Limitations of Study** 34](#__RefHeading___Toc190847571)

[**Anticipated Products/Interventions and Use** 34](#__RefHeading___Toc190847572)

[**Notifying Participants of Study Findings** 35](#__RefHeading___Toc190847573)

[**Dissemination of Results to Public** 35](#__RefHeading___Toc190847575)

[**VI. REFERENCES** 36](#__RefHeading___Toc190847576)

[**VII. APPENDICES.** 39](#__RefHeading___Toc190847577)

[**A. Online Intervention Pilot / Formative Work** 39](#__RefHeading___Toc190847578)

[**B. Survey Banner** 42](#__RefHeading___Toc190847579)

[**C. Informed Consents** 43](#__RefHeading___Toc190847580)

[**D. Description of Assigned Branch Conditions** 48](#__RefHeading___Toc190847582)

[**E. Baseline and Follow-up Survey (same instrument)** 49](#__RefHeading___Toc190847583)

[**F. Post-intervention Intent Questions** 51](#__RefHeading___Toc190847585)

[G. Example of Survey Exit Page 52](#__RefHeading___Toc190847587)

**Online Behavioral Interventions to Increase HIV disclosure and testing**

# I. PROJECT OVERVIEW

The purpose of this project is to develop and test the efficacy of online HIV risk reduction interventions among men who have sex with men (MSM). Since 2002, Public Health Solutions (formerly Medical and Health Research Association of New York City, Inc) has worked collaboratively with researchers on using the Internet as a recruitment tool for studies of high-risk sex and drug-using behaviors in MSM and, more recently, as an intervention venue. The primary objectives of the proposed behavioral interventions are to identify and engage MSM who are sexually active, in order to increase HIV disclosure with new male sex partners, HIV testing, and condom use. The interventions will be conducted by Public Health Solutions and extend prior online intervention research.

## Protocol Summary

The study was proposed by and extends prior research of Public Health Solutions (please see Appendix A). This protocol describes an Internet-based, five-branch randomized controlled intervention, enrolling 3,000 adult MSM. The five branches include 1) a control condition receiving no intervention content, 2) a standard text-based prevention webpage (from CDC.gov site: http://www.cdc.gov/hiv/topics/msm/index.htm), 3) an 8 minute dramatic video addressing sexual risk reduction within the context of alcohol use (The Morning After), 4) a 5 minute documentary video addressing HIV disclosure, HIV testing, and condom use with the sub-context of drug use (Talking About HIV), and 5) both videos. After completing the baseline survey, eligible participants who consent to participate in the intervention will be randomly assigned to the control condition or one of the four intervention conditions. The control condition will not include the delivery of any intervention content. All participants (branches 1-5) will be asked to complete a brief post-intervention survey (to measure intentions). For those in the control condition, the intentions survey will take place immediately after consent. After the intentions survey, all participants will be automatically directed to an exit page containing links to health related web sites and hotlines. Finally, all participants will be invited via email to complete a final 60-day follow-up survey.

## Study Site

## The implementation of the online intervention will be conducted on-site at Public Health Solutions. This RCT will be registered with clinicaltrials.org after IRB approval.

## Investigators/Collaborators

| Investigator/Collaborator. | Funding Mechanism | Federal wide  Assurance # | Research Engagement  Status |
| --- | --- | --- | --- |
| Sabina Hirshfield, PhD (PI) | Cooperative agreement | 00000489 | **Engaged** |
| Mary Ann Chiasson, DrPH | cooperative agreement | 00000489 | **Engaged** |
| Mike Humberstone | cooperative agreement | 00000489 | **Engaged** |
| Robert Remien, PhD | N/A |  | Not engaged |
| Francine Shuchat Shaw, PhD | N/A |  | Not engaged |
| Christopher Murrill, PhD | N/A |  | Not engaged |

CDC Project Staff

Andrew Margolis, MPH, PRB, DHAP, NCHHSTP, Atlanta, GAq

Heather Joseph, MPH, PRB, DHAP, NCHHSTP, Atlanta, GA

The CDC team is primarily responsible for providing technical assistance as needed in intervention development and in the design and conduct of research; ensuring that the intervention addresses empirically verified correlates of high-risk behaviors within the current study population; assisting in the development of the research protocol for CDC and local IRB review; assisting in designing a data management system, including coordinating data submission to CDC through the Secure Data Network (SDN) and developing cleaned data sets; working collaboratively with investigators to facilitate appropriate research activities; and analyzing data and presenting findings at meetings and in publications.

# II. INTRODUCTION

## Literature Review and Justification for the Study

## A resurgence in HIV transmission among men who have sex with men (MSM) is a cause for serious concern, as the number of newly diagnosed HIV infections among MSM increased 8% between 2003 and 2004.^1^ New HIV infections in MSM have been attributed to a number of behavioral factors including, but not limited to, safer sex fatigue, HIV treatment optimism, methamphetamine and other drug use, and easy access to sex partners through Internet hook-up sites.^2^ Studies have documented that MSM use the Internet for dating and sexual purposes.^3-11^ In fact, a recent meta-analysis of studies of online sex-seeking among MSM found that 40% of MSM used the Internet to seek sex partners.^12^ MSM have a considerably higher HIV prevalence rate than that of the general population, with reported sexual risk behaviors that include multiple sex partners,^13, 14^ unprotected anal intercourse (UAI),^15-18^ and alcohol and drug use, such as methamphetamines, Ecstasy (MDMA), gamma hydroxy butyrate (GHB), and ketamine.^14, 19-22^ The majority of research on HIV and risk behaviors among MSM has been conducted in small geographic areas or within large metropolitan cities,^23^ and there may be differing levels and correlates of drug use and sexual risk across geographic regions,^23^ contributing to or resulting in changing epidemic trends. The internet is a viable solution to reaching men across geographic regions and assessing drug use across different online populations.

The population of interest for this online intervention is MSM. Of MSM who have participated in online research, a considerable proportion of those who seek sex online may be particularly in need of Internet-based HIV prevention. They are well-educated, insured, and less likely to be exposed to offline prevention messages. ^5,24-26^ While online behavioral interventions have been developed and implemented successfully for treatment of certain conditions, such as depression,^27^ few have specifically targeted high-risk sexual behavior of MSM who meet men online. Even fewer have demonstrated the success of their methods (which have included chat room interactions around HIV and safer sex, and banner ads to promote HIV and STI testing).^28, 29^

A recent Public Health Solutions (formerly Medical and Health Research Association of New York City) online HIV behavioral intervention pilot successfully recruited participants online and reduced HIV risk behaviors. Men were 3 times more likely to ask their partner’s HIV status (OR=3.0, p<.001) and two times more likely to disclose their own HIV status (OR=2.2, p<.001) at 3-month follow-up compared to baseline. Additional information on pilot work and other background data can be found in Appendix A.

The proposed interventions and study design are significantly different from the pilot project, given the 5-branch, randomized control trial design. It also includes the ABC components (promotion of abstinence, faithful monogamy, and correct and consistent condom use) of HIV prevention. To the best of our knowledge, a study design evaluating the efficacy of multiple intervention approaches such as these has never been implemented or tested online with men seeking male sex partners.

**Intended/Potential Use of the Study Findings**

A recent study comparing the cost-effectiveness of HIV prevention interventions found that the two most important factors that determined cost-effectiveness were the HIV prevalence of the target population and the cost per person reached.^30^ In fact, one of the two most cost-effective interventions overall was showing videos in STI clinics (individual-level).^30^ These findings support our proposed online behavioral intervention using video vignettes in terms of cost-effectiveness and reaching high-risk MSM. If successful, this type of novel Internet-based intervention approach will be efficient, cost-effective, feasible to replicate, and especially useful in reaching men in rural areas or other hard-to-reach populations.

Specific Aims and Hypotheses

**Aim 1:** To test the feasibility of implementing an innovative national online randomized controlled, multi-branched, behavioral intervention for men who have reported oral or anal sex with any male (new partner or not) in the past 60 days AND reported oral, anal or vaginal sex for the first time (new partner) with a male or female within the past 60 days.

**Aim 2:** a) To identify and recruit men who have sex with men met online or offline, and who, in the last 60 days, met a new sex partner; b) To enroll and randomly assign 3,000 MSM, representing diverse race/ethnicities and geography, into one control or four intervention conditions; c) To maximize retention 60-days post-intervention to complete the follow-up survey.

**Aim 3:** To evaluate the intervention conditions by comparing outcome risk measures (HIV disclosure, HIV testing, condom use, drug and/or alcohol use before sex, and depressive and/or anxiety symptomatology) at 2 time points for 5 study conditions.

**Aim 3 Primary Hypotheses:** Between baseline and 60-day follow-up there will be an increase in the following: a) HIV disclosure with new sex partners, b) HIV testing, and c) condom use in intervention branches 3, 4, and 5, compared to branches 1 and 2.

**Aim 3 Secondary Hypotheses:** Between baseline and follow-up, there will be a decrease in the following: a) drug use before sex, b) alcohol use before sex, and c) depressive and/or anxiety symptomatology in intervention branches 3, 4, and 5, compared to branches 1 and 2.

# III. PROCEDURES / METHODS

**Study Design and Location**

This Internet-based, five-branch randomized controlled intervention trial, will aim to recruit men who reside anywhere in the United States. However, the study will be managed by scientists located at Public Health Solutions in New York, NY.

**Procedures, Duration, Experimental Procedures**

We hypothesize that a brief online behavioral intervention eliciting critical thinking and attitude and behavior change will yield a reduction in sexual transmission risk, and increase HIV testing and HIV disclosure to sexual partners among MSM. By incorporating multiple approaches to online intervention content and delivery, we will test the efficacy and relative effectiveness of 4 (intervention) study conditions to improve primary outcomes of interest. The study conditions are summarized as follows:

| Branch | Description |
| --- | --- |
| 1 | control condition (i.e., shown exit page* with health links, but no intervention content) |
| 2 | standard text-based prevention webpage (http://www.cdc.gov/hiv/topics/msm/index.htm); |
| 3 | 8 minute dramatic video addressing sexual risk reduction within the context of alcohol use (*The Morning After)* |
| 4 | 5 minute documentary video addressing HIV disclosure, HIV testing, and condom use with the sub-context of drug use (*Talking About HIV*) |
| 5 | Both videos |
| *Note: all five branches will have the same list of resources available to them on the survey exit page, which will provide links to HIV testing, drug and alcohol treatment, mental health hotlines, and information about abstinence, mutual monogamy, and consistent condom use. | |

**Evaluation of Branches**

Participants in all branches will be scheduled to complete three quantitative surveys including a baseline assessment, a brief immediate post-intervention assessment (or immediate post-baseline assessment for the control group) measuring intent to change behavior during the next 60 days, and a 60-day follow-up assessment. Additionally, we will record the number of “clicks” on the resources webpage (presented after the post-intervention assessment) which represent intent to access health services and information. Our 5-branch design will allow us to directly compare, within and across branch, changes in primary and secondary outcome behaviors from baseline to 60 days follow-up. By doing so, we will be able to measure the efficacy of each of the study branches, as compared to the control branch and standard text-based prevention branch and relative to each other. The multi-branch design will allow us to directly compare the video-based interventions to the text-based prevention web-page to determine which branch elicits the most significant change in behavior.

**Theoretical Framework for Standard Text-Based Prevention Web Page**

***Branch 2:*** Internet-based information about HIV may be a useful medium for primary prevention.^3^ The standard text-based prevention web-page is located on the CDC.gov website (http://www.cdc.gov/hiv/topics/msm/index.htm), which contains facts about HIV among MSM, with sidebar links to prevention information, resources, publications, and other information pertaining to HIV. We will be able to time-stamp how long the participant was on the CDC web-page, based on the time between entering and exiting the web page.

**Theoretical Framework for Video Interventions**

***Branches 3-5:*** Media presentations that possess emotional qualities yield internal reflection and critical thinking,^31^ and there is growing evidence that dramatic or documentary narratives are more influential than written text.^32^ A major requirement for dramatization to be effective is that it creates an emotional response to persuade the viewer to accept the intended message.^33^ Film and video, in comparison with text, more often invoke “participatory responses,” essential to extended thoughts about characters, expressions of preferences about events, reflections on the broader implications of the content.^34, 35^ The use of film or video to create narratives, stories, or case studies, whether dramatic fiction or documentary has a powerful and enduring effect on critical thinking, attitude, and behavior change. Thus, videos are richer, more compelling, and more memorable than written text.^36^

The **dramatic video** (*The Morning After*, described in Appendix A) and the **documentary video** (*Talking About HIV*, described below) are based on the same theoretical framework in relation to the goal of this intervention. Both video treatments share common learning goals and the same target audience of learners. Both address the issues of sex and disclosure, condom use, and substance use (with references to HIV testing) among MSM realistically. However, they represent realism very differently. *The Morning After* is designed as a scripted drama and a fictional story with a carefully crafted plot and characters based on reality. *Talking About HIV*, excerpted from a cinema verite documentary (*Meth*), includes authentic subjective accounts, first-hand stories, and testimonials told by individuals about their own experiences.

*Talking About HIV* has been created from director Todd Ahlberg’s (2005) documentary film, *Meth*. In *Meth*, current and past methamphetamine-using gay men share their experiences about the drug’s effect on their lives, relating to meeting sex partners, HIV disclosure, HIV infection, condom use, and the negative impact of substance use on health, family life, employment, and housing. We have developed a 5-minute documentary from the video footage, which focuses on HIV disclosure, references to HIV testing, and condom use.

The theoretical framework for this study is tailored to the objectives of promoting critical thinking and attitude and behavior change, precursors to the broader goals of increasing HIV disclosure, HIV testing, and condom use among men who have sex with men. The two video treatments in this study, though intentionally different in genre and degree of verisimilitude, are designed to challenge viewers and prompt critical thinking by portraying (and providing the vicarious experience of) dissonance, disequilibrium, and expectation failure through the instructional strategy of modeling. *The Morning After* is contextualized as a dramatic story and *Talking About HIV* is contextualized as personal accounts of stories and subjective testimonials.

Dissonance and disequilibrium both involve the discomfort felt when there is a discrepancy between what an individual already knows or believes and new information or interpretations encountered. Cognitive dissonance theories^37^ hold that such contradicting cognitions, and their attendant affective dimensions, are typically aroused when an individual actually needs to accommodate new ideas. When a new experience, piece of information or idea encountered does not fit well with existing knowledge and understandings, individuals experience disequilibrium.^38^ This frame of mind then serves as a driving force for change, creating a window of opportunity to open up to new ideas. Realistic and testimonial stories can leverage these related instructional strategies, through modeling.

One critical phase in this learning process is “expectation failure.” That is, from a cognitive science perspective, what these theories share is the belief that learning, and in particular learning critical thinking skills, is stimulated by failure. Schank^39^ states that “*This* is the opportunity to learn . . . Individuals realize that something was wrong with the original, existing perception of how the world works, that failure resulted from a faulty representation of something in the goal domain.” Expectation failure can be modeled, used as the basis for structuring the content in realistic stories with which viewers can identify, to stimulate critical thinking and attitude and behavior change. Thus, stories provide powerful vicarious experiences for critical thinking and attitude and behavior change.^40^ Both video treatments in this study attempt to set the stage for this teachable moment.

Essential to modeling, the instructional strategy associated with observational learning, is the portrayal of imperfection, flawed thinking, performance failure, problematic behavior for the viewer’s vicarious experience. As Bandura^41-44^ points out, we learn very little from a “perfect performance”; rather, it is from the exposure and identification of problems, and the reasoning-through-the-resolution process, that individuals are far more likely to learn. Models most effective for learning *make present* (in picture and sound) their goals and incentives; critical choices or decision points, and related strategic thinking, questioning, actions and consequences. Effective models experience trial and error; that is, effective models reveal to viewers the process of learning through failure. Models should demonstrate humanity -- frustration, self-control, effort, struggle, remorse, satisfaction, as appropriate throughout.

**Audience/Stakeholder Participation**

The primary audience for this project is men who have sex with men recruited online who have reported oral or anal sex with any male (new partner or not) in the past 60 days AND reported oral, anal or vaginal sex for the first time (new partner) with a male or female within the past 60 days. This internet-based project inherently provides a mechanism to deliver intervention content broadly, and as a result, can directly benefit multiple governmental and community-based stakeholders committed to improving public health. We have already seeked specific guidance and feedback on intervention and evaluation content from our established community advisory board (the Internet-based Intervention Advisory Group, comprised of members of public health agencies and the online community). These individuals have also pilot-tested and commented on the baseline and follow-up surveys, as well as the text and video content.

**Risks to Respondents**

Participation is voluntary and confidential. The research involves minimal physical and social risk to participants. It is possible that participants may feel embarrassment or discomfort with the intervention videos, the surveys, or both. The only foreseeable risk would be a breach of confidentiality if participants’ email addresses were inadvertently released and linked to their survey responses.

**Protection against Risks**

Intervention and research materials will be reviewed and approved by the Institutional Review Boards (IRBs) of Public Health Solutions and the CDC prior to beginning study activities. In the unlikely event of a participant becoming distressed from answering the survey or watching the intervention videos, we will have a link to a 24-hour, toll-free mental health hotline (LifeNet: 800-273-8255) on the exit referral page. Participants will see contact information about the local IRB and investigator to phone or email if they have any concerns or questions or experience any negative impact. Any adverse events will be reported to both Public Health Solutions and CDC IRBs.

Informed Consent: A printable online consent form will describe the study to the potential participant following a click of the study recruitment banner (please see Appendix C-1). Participants who do not wish to complete the baseline survey will be able to discontinue their participation by closing their Internet browser and will have the option of declining to answer any questions that they do not wish to answer. Those who complete the baseline survey but do not meet intervention study criteria will automatically be directed to the exit page, which contains links to HIV testing, drug and alcohol treatment, mental health hotlines, and information about abstinence, mutual monogamy, and consistent condom use (Appendix G). Those who complete the baseline survey and do meet intervention study criteria will be provided with another consent form (Appendix C-2) asking them to join the next part of the study that involves assignment to one of five branches and an additional 60-day follow-up interview. This consent also specifies that it is necessary to provide an email address to participate. The minimal risks, the confidential and voluntary nature of participation, and the fact that participants can stop their participation at any time are described in the consent form. Finally, before respondents begin the 60-day follow-up survey, they will be shown a reminder (Appendix C-3) that highlights the information presented in the second consent. See the *Informed Consent* section below for more details about these procedures.

Email addresses and data security: An email address will be the only identifying information collected; however, the email address will be encrypted and stored separately from survey data. Email addresses will be stored in an electronic file separate from survey data on a secure, firewall protected Public Health Solutions-managed server. Only the Public Health Solutions Manager of Web Development and Programming will have access to these files. If email addresses were inadvertently released, they would be un-readable. After all of the 60 day follow-up surveys have been completed, the encrypted email addresses will be destroyed. For those respondents who do not respond after 3 email follow-up attempts, we will delete the email address when their follow-up window expires. Other than email address, no other personally identifying information will be collected. No client-side persistent cookies will be used. For example, no cookies will be left on the user’s computer between the time of baseline and 60-day follow up.

Data will be stored on the Public Health Solutions, Data Link password protected server and will not be accessible by any persons who are not directly involved in the research. The server is protected by firewalls and other safeguards. Only members of the research team will have access to the data. Data will be backed up regularly and stored securely according to Public Health Solutions disaster recovery and business continuity protocols.

**Potential Benefits**

All respondents will have the opportunity to view web links to HIV/STI, drug, alcohol, and mental health treatment at the end of both surveys and will be given an option of writing (either anonymously or by providing an email address for a response) their reactions to the survey and directing questions to the principal investigator (Dr. Hirshfield). We successfully used this format in all of our prior online studies. Only a small proportion of participants (about 1%) have chosen to directly contact the investigators.

Benefits to society in general are anticipated through the dissemination of intervention findings (i.e., regarding high-risk sexual behavior and drug use). Results will inform local and national public health agencies about the magnitude of these problems and help them to develop outreach and prevention strategies that target Internet users, as well as the local community. Potential benefits of this type of Internet-based intervention approach include its efficiency, cost-effectiveness, and ability to be replicated.

Study Timeline

| **Date** | **Deliverables** |
| --- | --- |
| 10/06 – 05/07 | Develop Intervention Protocols |
| 10/06 – 05/07 | Develop Instruments (with consensus between CDC and Sites) |
| 10/06 – 05/07 | Develop Data Management/Sharing Plans |
| 12/06 – 06/07 | Develop Intervention Materials (e.g., videos, web pages) |
| 05/07 – 06/07 | Work with CABs/expert Advisory Groups |
| 07/07 | Submit IRB Packages to Prevention Research Branch |
| 07/07 – 09/07 | Submit IRB Packages & Obtain Approvals (Local and CDC) |
| 09/07 – 10/07 | Implement Pilot Intervention Testing |
| 10/07 – 11/07 | Fine-Tune Intervention |
| 12/07 | Submit IRB Amendments & Obtain Approvals |
| 1/08 – 5/08 | Recruit & Enroll Intervention Participants and Implement Intervention |
| 5/08 – 10/08 | Follow-up Participants (60 days post-baseline) |
| 10/08 – 12/08 | Data Cleaning & Verification |
| 01/09 – | Data Analysis |
| 01/09 – | Disseminate Findings |

**Internet Banner Ad Clicks (N=19,486)**

**Meet Criteria for Intervention Inclusion (49%=4,125)**

**Consent to participate in baseline survey (54%=10,522)**

**Complete Baseline Survey** **with Programmed Screening Questions (80%=8,418)**

**Computerized Randomization**

**YES**

NO

Survey Exit Page

**YES**

NO

Survey Exit Page

**Attrition 30%~420**

**Attrition 30%~420**

**Branch 2**

**20%=600**

**Branch 3**

**20%=600**

**Branch 4**

**20%=600**

**Attrition 30%~420**

**Branch 1**

**20%=600**

**Control**

**60-day follow-up survey**

**Attrition 40%~360**

**60-day follow-up survey**

**60-day follow-up survey**

**60-day follow-up survey**

**Complete Post-Intervention Survey (Intentions to Change Behavior)**

**Attrition 40%~360**

**Standard Prevention Webpage**

**‘Morning After’ Video**

**‘Talking About HIV Video’**

**Both Videos**

**Branch 5**

**20%=600**

**60-day follow-up survey**

**Consent to give email and be followed (73%=3,000)**

**Study Design for 5-Branch Randomized Controlled Trial**

Number of Online Ad Impressions Potentially Viewed = 847,217

Model estimates are based on a previously conducted online survey of MSM.^26^ The click-through-rate (CTR) was 2.3% on Manhunt.net, yielding the Banner Ad Click estimate (clicks/impressions=CTR).^45^ The estimate for consenting to give an email address and participate in the intervention is based on the online pilot intervention.^46^

**STUDY POPULATION**

## General Description

### The overall study population will consist of approximately 10,500 MSM. The study sample for the intervention (those randomly assigned to branches 1-5) will be 3,000 MSM who reside in the United States, are age 18 and over, who have reported oral or anal sex with any male (new partner or not) in the past 60 days AND reported oral, anal or vaginal sex for the first time (new partner) with a male or female within the past 60 days, and who represent diverse race/ethnicities and geography.

## Intervention Participant Inclusion Criteria

• Male;

• Age 18 and over;

• Ability to read and respond in English;

• Reside within the United States;

• Report oral or anal sex with any male (new partner or not) in the past 60 days AND reported oral, anal or vaginal sex for the first time (new partner) with a male or female within the past 60 days;

• Complete baseline survey

• Provide an email address;

The recruitment web site will be a subscription-based adult site that requires users to confirm that they are 18 years or older when registering. The study consent form explicitly states that men must be 18 or older to participate. However, this is an internet-based study and therefore the study team will be unable to verify the validity of inclusion criteria, including age.

The only inclusion criteria that will be shared with participants (in the consent form) is gender, age, residence in the U.S., and “sex with another man.” The detailed behavioral inclusion criterion for participation in the intervention trial will be measured in the context of the baseline survey. The likelihood of potential participants learning the criteria and returning to the site to retake the survey will be minimized. The research team will be able to identify duplicate email addresses and determine appropriate action during the data cleaning phase of the project. However, the research team does not anticipate that this will be a significant issue given that study activities represent time away from an individual’s recreational internet activities.

## Intervention Participant Exclusion Criteria

• Women and transgender persons

**Exclusion of Women and Transgender Persons**

The purpose of our online intervention is to reduce sexual risk and drug and alcohol use among MSM; therefore, women are excluded from the intervention. Women and male-to-female transgender respondents will automatically be skipped to the last section of the baseline survey on general health. Female-to-male transgender respondents will be able to complete the baseline survey but will not be eligible for the intervention study. No transgender participants will be eligible to participate in the trial since this study is specifically for biologically male participants.

**Exclusion of Children**

This intervention involves men age 18 and over. The NIH definition of children is anyone under the age of 21.^47^ However, the Public Health Solutions IRB does not permit recruitment of participants under age 18 in online surveys. Additionally, children (those under the age of 18) are excluded from participation because they are likely to be at different cognitive and developmental stages than young adults. Thus, an intervention for minors would necessitate a different intervention approach tailored for their developmental age. Finally, the gay websites we will partner with have a policy against registering anyone under age 18.

If an individual under the age of 18 attempts to participate in the baseline and enters his true age in the assessment, he will automatically and immediately be skipped to the referral page at the end of the survey, which will provide links to HIV testing, drug and alcohol treatment, mental health hotlines, and information about abstinence, mutual monogamy, and consistent condom use.

**Estimated Number of Participants**

The following estimates are based on our previous research (Appendix A). We expect that 10,522 individuals will consent to participate in the baseline behavioral assessment. Eighty percent of these individuals (8,418/10,522) will likely respond to all of the items that assess eligibility for participation in the larger intervention study. Forty-nine percent of these individuals (4,125/8,418) will likely meet the criteria for intervention inclusion, and 73% (3,000) will likely consent to participate in the intervention evaluation study.

**Sampling, Sample Size, and Statistical Power**

The three primary outcomes in this study are increased HIV testing, HIV disclosure, and condom use. Using historical data from the pilot intervention (see Appendix A), we observed that HIV testing increased from 20% to 42% in HIV-negative men between baseline and 3-month follow-up. Based upon these values using a 2-group design (control vs. intervention), with a 5% significance level and 80% power, 77 participants would be required in each study condition for a total sample size of 154. In the current study, which has a 5 branch design, we anticipate that by enrolling 3,000 men and retaining 70% (N=2,111), we will have sufficient power to detect meaningful differences in behavior among the study branches.

Although we will be comparing within and across all study branches, we expect the greatest differences to be found between control branch 1 and branch 5 (both videos) for the primary outcomes. The Bonferroni correction will be used to protect the overall Type I error rate with multiple comparisons. In these comparisons, the nominal significance level will be set to alpha = 0.05 for two-sided tests (divided by the number of pairwise comparisons for individual tests). Using Chi-square tests to detect behavioral changes between the binomial primary outcomes, we obtain the following powers to detect a difference between two study branches for various true proportions and sample sizes (α=0.05; 10 pairwise comparisons).

| **Power Calculations for Meaningful Differences**  **between Study Branches** | | | | | |
| --- | --- | --- | --- | --- | --- |
| **Behavior Change Outcomes†** | | **Sample Size per Branch* and Associated Power** | | | |
| Branch A | Branch B | N = 300 | N = 400 | N = 500 | N = 600 |
| 0.10 | 0.15 | 0.142 | 0.221 | 0.308 | 0.396 |
| 0.10 | 0.20 | 0.709 | 0.865 | 0.944 | 0.979 |
| 0.10 | 0.25 | 0.978 | 0.997 | >.999 | >.999 |
| 0.15 | 0.20 | 0.097 | 0.150 | 0.210 | 0.275 |
| 0.15 | 0.25 | 0.569 | 0.747 | 0.863 | 0.931 |
| 0.15 | 0.30 | 0.939 | 0.988 | 0.998 | >.999 |
| 0.20 | 0.30 | 0.476 | 0.652 | 0.784 | 0.874 |
| 0.20 | 0.35 | 0.894 | 0.971 | 0.993 | 0.999 |
| 0.20 | 0.40 | 0.994 | >.999 | >.999 | >.999 |
| 0.30 | 0.40 | 0.375 | 0.536 | 0.673 | 0.780 |
| 0.30 | 0.45 | 0.821 | 0.936 | 0.980 | 0.994 |
| 0.30 | 0.50 | 0.984 | 0.998 | >.999 | >.999 |
| 0.40 | 0.50 | 0.336 | 0.488 | 0.623 | 0.733 |

†Behavior change outcomes refer to the primary outcomes of interest: HIV disclosure, testing, and condom use.

*We anticipate enrolling 600 per branch; however, attrition may yield smaller sample sizes.

**Participant Recruitment and Enrollment Procedures**

This study will be carried out by Public Health Solutions with the cooperation of one gay-oriented website (e.g., www.manhunt.net), and possibly additional gay-oriented websites (i.e., bigmuscle.com, rentboy.com, and blackgaychat.com) if enrollment figures are not being adequately achieved. All participants in the baseline survey (approximately 10,500) are considered part of the study population and, regardless of whether they are included in the intervention or not, will be included in descriptive analyses. Although one web site is projected as being adequate to meet study goals (N=3,000 randomized into the study), multiple websites may have to be utilized if recruitment is lower than anticipated. A banner ad placed strategically throughout the participating web sites (e.g. chat rooms, exit page) will draw attention to the study. This online recruitment method is considered passive, as opposed to actively recruiting men in chat rooms to take part in the study. Those who click on the recruitment banner (example in Appendix B) will see the first of three consent forms. The first consent form (Appendix C-1) will describe the study in general terms. We will provide our email and phone number contacts here to address further questions about the study. All potential participants will be given the opportunity to exit the site if they do not wish to continue. Following completion of the first informed consent process, participants will be administered the online baseline behavioral survey (Appendix E).

All potential intervention participants who complete the baseline survey, meet study criteria, and provide consent (explained in the second informed consent document) (Appendix C-2) will automatically be randomized into the intervention. After study condition assignments are made, each participant will see one page that describes the assigned condition in more detail (see Appendix D). However, participants in branch 1 will not see anything after the baseline survey other than the post-intervention questions and the resource page that all participants will be shown. Participants in branch 2 will view a text-based standard prevention web-page that contains text and graphics. Participants in branch 3 and 4 will view one of two short videos and those in branch 5 will see both. All participants will then complete a short post-intervention survey (Appendix F) assessing behavioral intentions.

Participants who drop out of the baseline survey, do not answer questions that assess eligibility, or who drop out before randomization will be considered non-responders. Men who are found ineligible for the intervention component of the study will be shown a script such as “Thank you for your time. We appreciate your input in this study. Here are some links to helpful websites about staying healthy.” Participants will then be shown the survey exit page (example in Appendix G), which will provide links to HIV testing, drug and alcohol treatment, mental health hotlines, and information about abstinence, mutual monogamy, and consistent condom use (ABC). For those who are eligible, the second consent form (Appendix C-2) will be presented immediately after the baseline.

All participants will be scheduled for an online follow-up survey 60 days post-intervention, at which time they will receive an email with a hyperlink to the follow-up. A study reminder (Appendix C) will precede the 60-day follow-up survey (Appendix E) and will contain similar information to the second consent. After reading the reminder, participants will choose whether they would like to continue to the survey or not. Regarding intention to treat (ITT), once participants consent and are randomized, they will be kept in their original assignment group and be sent a link to the 60-day follow-up survey even if they do not participate in the intervention activity (i.e., watch video(s) or view standard prevention web page) or answer the post-intervention questions.

**Randomization Procedures**

The goal is to have a randomly assigned study sample of approximately 3,000 participants in five groups balanced within a 5% range. Since chance may not lead to balance, we developed a type of blocking that preserves random assignment while assuring a balanced sample through the duration of the study.

Using the server clock, the decisecond (0,1,2,3,4,5,6,7,8 or 9) when a participant clicks their mouse to go to the “branch” page of the survey is captured.

This “draw” is mapped to the 5 branch groups (a draw of 5-10 is “re-drawn” until a 0-4 comes up), giving us a random draw of 5 numbers. Each half-second, a participant has a 1 in 5 chance of being assigned to any given branch.

When 100 participants have been through the draw, we begin an evaluation procedure to determine the balance among the groups that has been achieved by chance. This process:

- Counts the number assigned to each group
- Determines each group’s percentage of the whole
- Designates each group as:
  - “high,” meaning it contains more than 22.5% of the participants
  - “low,” meaning it contains less than 17.5% of the participants
- Determines if the block (100 records at this point) is “balanced” or “not balanced” within the objective 5% range

If a group (or groups) is “high,” that number is taken out of the “draw” until that group no longer represents more than 22.5% of the whole. This means that during this condition, participants are being randomly assigned to the remaining groups. If one group is “high,” participants have a 1 in 4 chance of being assigned to each of the other groups.

If a group (or groups) is “low,” that number is “emphasized,” meaning an additional number which results in an assignment to that group is added to the “draw” (from the unused 5-9), effectively increasing the chances of assignment to that group from 1 in 5 to 1 in 3. If two groups are emphasized, the chances increase to 1 in 3.5. Again, assignments continue to be made to all groups randomly. (No more than two groups would ever be emphasized at the same time.)

This “nudging” up of low groups and down of high groups continues until the block becomes “balanced” with each group having between 17.5%-22.5% of the whole. Once “balance” is achieved, the next block begins, and the procedure starts again when the next 100 participants have been assigned.

The key point is that random assignment continues through the balancing process. This is preferable to fully preventing assignment to full groups while waiting for the others to “fill up,” which could result in the last group to fill being the only assignment option for a length of time, risking bias and exposing the survey to temporal factors.

In testing, some blocks were balanced at 100 participants. Other blocks took additional participants to balance, resulting in a random range of block sizes. The average block size in tests of a 3,000 participant simulation was 133 records.

**Attrition and Retention Efforts**

Sample selection procedures are designed to produce a sample of approximately 3,000 MSM (600 men per randomized branches 1-5) at the end of the recruitment period. Past formative work, which included piloting an internet-based intervention with a 90-day follow-up survey, showed an attrition rate of 45%, with slightly less than 500 of the 1,000 men who provided emails completing the follow-up survey. Given the shorter follow-up period (60 day follow-up) in this research study, we expect a lower overall attrition rate and a differential attrition rate by branch. We estimate that control branch 1 and standard text-based prevention web page branch 2 will yield an approximate attrition of 240 (40%) participants, and video treatment branches 3-5 will yield an approximate attrition of 180 (30%) participants. We are expecting higher attrition for branches 1 and 2 since there is no video component, which is more engaging.

We will make efforts to minimize study attrition by sending up to 4 reminder emails (Appendix C-3) to participants during the course of the follow-up period. For participants who do not respond to the follow-up email, we will wait approximately 1 week before sending a first reminder email, approximately another week before sending the second reminder, and approximately one more week before sending the final reminder email. In our previous work, virtually no additional responses were obtained after 3 reminders.

**Incentives**

Research evaluating behavioral surveys administered over the internet has found that offering incentives likely increases the proportion of participants who enroll into a single study multiple times to maximize their ability to receive monetary reimbursement.^48^ In an effort to avoid or reduce the proportion of individuals who repeatedly enroll into this study, participants will not receive monetary reimbursement for completing study activities.

**Informed Consent Procedures**

The informed consent process for this study is in 2 parts: 1) Participants who click on a banner ad will be required to review and complete the online informed consent by clicking a button that indicates they read the consent form and wish to participate in the baseline survey. The consent form will state that the study is limited to persons aged 18 or older, for men who have sex with men, and addresses substance use and sexual risk behaviors associated with HIV transmission. Men who provide informed consent by clicking the consent form button will be allowed to enter the online baseline survey.

2) If the participant meets criteria for the intervention study (which will be automatically programmed to identify eligible participants), he will see another consent form that will invite him to participate in a brief intervention and ask for his email address. He will be informed that the study is randomized, and a brief description will be given for the study branches. If the participant consents by clicking the consent form button, then he will automatically be randomized into one of the assigned conditions. Before the participant begins the intervention, he will see information that clearly describes the assigned condition (i.e., prevention web page or video). For those who are assigned to the control condition, a screen will appear with the post-intervention questions, and then they will see a statement about being contacted in 60 days for a follow-up survey.

Intervention participants will be contacted via email 60 days post-intervention to complete the follow-up survey. A hyperlink will be embedded in the email, and when clicked on, will automatically transfer the participant to the consent reminder, which highlights the same information from the second consent. Men are given the opportunity to go on to the next page to begin the survey or to close their browser. Participants will be able to print copies of the consent forms and the reminder.

For participants who do not respond to the follow-up email, we will wait approximately 1 week before sending a first reminder email, approximately another week before sending the second reminder, and approximately one more week before sending the final reminder email (for an additional total of 21-30 days). The maximum number of days for completion of the follow-up survey will be 75 days.

**Justification to Waive Documentation of Consent**

Although Federal regulation requires that researchers obtain written informed consent for research on human subjects, under 45 CFR 46.117(c) written consent can be waived for research that involves minimal risk to participants and involves no procedures for which written consent is normally required outside of the research context.^49^ This research meets that criterion. We propose an alternative approach where participants click a button, signifying that they have read the informed consent page and agree to participate in the study. An advantage of Internet-based studies is that the consent form is available for the subject to review at any time.^50^ This strategy complies with the requirement of 46.117(c) that participants are given a written statement describing the research and risks.

**Study Instruments and Variables**

Respondents from all study branches will have the opportunity to complete baseline, post-intervention and follow-up assessments, which based upon previous piloting activities, each average about 10 minutes in duration. Survey questions have been adapted from questionnaires used by the research team in their previous online studies and pilot intervention. The online baseline and follow-up questionnaires will include information on the following domains:

- demographics (year of birth, race/ethnicity, education, income and the first 3 digits of the US zip code)

- depression and anxiety symptomatology

- assessment of risk behaviors, such as type of sexual contact (anal, oral, vaginal -- with and without condoms)

- knowledge of partners’ HIV status and disclosure of participants’ HIV status

- drug and alcohol use (in general and use before or during sex)

- how sex partners were met (online versus other ways)

- HIV and STD testing

- knowledge of HIV status

Please see ‘Additional Outcome Variables’ under Primary and Secondary Outcome Variables below. All surveys are located in the Appendices.

**Standardized Measures Included in Instruments**

Depression and Anxiety Symptoms: The Patient Health Questionnaire for Depression and Anxiety (PHQ-4)^51^ is a four-item scale that will be used. The PHQ-4 comprises the PHQ-2, which assesses depressed mood and anhedonia, and the GAD-2, which assesses generalized anxiety disorder. The PHQ-4 begins with the stem question: “Over the last 2 weeks, how often have you been bothered by the following problems?” The four screening questions are: Feeling nervous, anxious, or on edge; Not being able to stop or control worrying; Feeling down, depressed, or hopeless; and Little interest or pleasure in doing things. Responses are scored as 0 (“not at all’), 1 (“several days”), 2 (“more than half the days”), or 3 (“nearly every day”). The total score on this composite measure ranges from 0 to 12 (the score range for both the PHQ-2 and GAD-2 is 0 to 6). Kroenke and colleagues measure depression and anxiety screens separately. Thus, if a participant scores 3 or higher on either the PHQ-2 or the GAD-2, then he will have screened positive for that mental health problem. A score of 3 is considered the optimal cut-point for screening purposes.^52, 53^

Reasons for Not Getting Tested: We will be using a slightly adapted version of the CDC sponsored HIV testing Survey (HITS), which inquires about the various reasons people don’t get tested. The instrument targets HIV-negative or untested individuals at-risk for HIV infection.

Defining Sex Partners: The definition of male or female main partner for this study is: Main Partner (boyfriend [or girlfriend], spouse, significant other, or life partner). This definition will be used for the 60 days prior to and after baseline. We will be able to determine new partners (first sex within the past 60 days) from the question, “When did you first have sex with xxx?” The response options are (‘within the last’) 24 hours, 7 days, 30 days, 60 days, 1 year, more than a year ago. If the respondent chooses 60 days or sooner, then we know this was a new partner.

**Primary and Secondary Outcome Variables: Baseline & (60-day) Follow-up Period**

These questions will be included in the baseline survey and the 60-day follow-up surveys

| **Concept** | **Number of Key Variables** | **Primary Outcome Variables** | **Measurement for Statistical Analysis** |
| --- | --- | --- | --- |
| HIV Disclosure: Asked partner(s); partners asked | 4 variables | Likert scale for “asking HIV status” by partner serostatus | (Ordinal), Treated as Continuous |
| HIV Disclosure: Told partner(s); partners told | 4 variables | Likert scale for “telling HIV status” by partner serostatus | (Ordinal), Treated as Continuous |
| HIV testing / Reasons for not getting tested | 10 variables | Number of times tested, last time tested and test result, if HIV negative or unknown will respondent plan to get tested and when? If never tested, reasons for not testing. If HIV-positive, when did they get test result (month/year). | Categorical |
| Current Condom Use | 8 variables | Condom use (receptive and insertive) with new and/or steady partners in past 60 days. | Categorical |

| **Concept** | **Number of Key Variables** | **Secondary Outcome Variables** | **Measurement for Statistical Analysis** |
| --- | --- | --- | --- |
| Drug Use | 15 drugs | Frequency of drug use and typical drugs (route of administration) used in past 60 days. | Continuous, categorical |
| Alcohol Use | 4 variables | Frequency of alcohol use and number of drinks drank (binge drinking) (5+ drinks) before/during sex and in general past 60 days. | Continuous, categorical |
| Depressive Symptoms, last 2 weeks | 2 variables | Positive Depressive Symptom Score: PHQ-2, part of PHQ-4. | Interval (Will be dichotomized) |
| Anxiety Symptoms, last 2 weeks | 2 variables | Positive Generalized Anxiety Disorder Score: GAD-2, part of PHQ-4. | Interval (Will be dichotomized) |
| **Concept** | **Number of Key Variables** | **Additional Variables** | **Measurement for Statistical Analysis** |
| Drug or alcohol treatment | 1 variable | 60-day drug or alcohol treatment (yes, no) | Dichotomous |
| Health seeking behavior | 2 variables | Clicks on treatment links on the survey exit page- do the proportion of clicks vary by intervention branch? | Dichotomous |
| Intention to Change Behaviors: Post-Intervention Assessment | 4 variables | Within the next 60 days… How likely are you to: use condoms with new sex partners? ASK your new sex partners their HIV status? TELL your new sex partners your HIV status? (if negative or untested) Get an HIV test? | Continuous |
| New Partners | 2 variables | Number of NEW male or female sex partners in past 60 days prior to baseline and 60-day follow-up. | Continuous |
| Diagnosis of Depression, Anxiety, and other conditions | 10 variables | Ever diagnosed – qualitative option for diagnoses not listed | Dichotomous |
| Past 60-day mental health counseling | 1 variable | If yes, past 60 days, or longer than 60 days ago. | Categorical |
| Current medications |  | Checkbox selection of medications plus “other medication” qualitative option for medications not on list | Categorical, string |

**Additional Measurements**

Immediately after the intervention activity (or baseline for those who are randomized to branch 1), participants will be asked to complete a brief intentions survey (Appendix F). These questions will focus on HIV disclosure, condom use, alcohol/drug use, and HIV testing (for those who are HIV-negative) in the next 60 days.

Additionally, we will also record the number and categories of link “clicks” that consented respondents make when shown the resource page after completing the post-intervention assessment (baseline) and after completing the follow-up assessment. This page, found in Appendix G, contains links to a variety of health related resources. The data will be automatically added to the individual participant’s data file.

**Outcomes and Minimum Meaningful Differences**

The primary outcomes are increases in HIV serostatus disclosure, HIV testing, and condom use between baseline and 60-days follow-up. Although we will be comparing within and across all branches, we expect the greatest differences to be found between control branch 1 and branch 5 (both videos) for the primary outcomes. A minimal meaningful difference for the primary outcomes will be to detect statistical significance in behavior change between baseline and follow-up at both the 0.01 and 0.05 alpha levels.

**Training for Study Personnel**

No additional special training is necessary for this intervention project. The PI and co-investigators are trained in this type of research and have been conducting internet-based research for the past 5 years. No interviewers are needed. The PI will be the individual listed as the primary contact should a participant require additional information or have concerns with the study. The PI has specific experience in this regard.

**DATA HANDLING AND ANALYSIS**

Although no personally identifying information other than email addresses (which will be encrypted and stored separately), will be collected at any point during the surveys, several technical steps will be taken to ensure the security and privacy of data in general. All survey data will be stored on a secure Public Health Solutions web server with firewalls and industry standard protections and will be removed from the web server at the end of the survey period.

All analyses will be performed in SPSS 14.0 and SAS 9.1 for Windows. For the overall sample (3,000) we will use a reduced alpha level (.01) as a criterion for statistical significance. For the within-branch analyses (n=600 or less) we will use the conventional alpha level (.05). Due to decreased power, all interaction tests will be conducted at the 0.10 level of significance.

The first analytic procedure will be to use ANOVA or chi-square to compare key characteristics (i.e., age, race/ethnicity, income) among and between the 5 randomized groups to ensure that there are no statistical differences, and to verify that computerized randomization worked.

**Frequency Distributions**: Frequency distributions will be produced for all variables and be used to describe the sample characteristics and answer key questions (e.g., what percentage of men reported unprotected anal or vaginal intercourse in their last sexual encounter with a new male or female partner?).

**Bivariate Analysis:** Bivariate analysis will be conducted to identify measures that show significant associations with primary outcome variables, (e.g., asking or telling HIV status to new sex partners [yes/no], HIV testing during the 60 days after baseline [y/n], and unprotected anal or vaginal intercourse [y/n]). Associations between measures with more than two values (e.g., drug use increased, decreased, or remained the same) and binary outcomes (e.g., HIV/STI testing [y/n]) will be tested, though the Pearson Chi-Square test and t-tests will be performed with continuous variables (e.g., number of sex partners in past 60 days).

**Multivariate Analysis:** For dichotomous outcomes, we will use logistic regression models to simultaneously examine the effects of predictors found to have a statistically significant association with the binary outcomes in bivariate analyses. Further, logistic regression will allow the introduction of the pretest observation (e.g. asked new partner’s HIV status) and treatment status as predictors and a set of demographic control variables. For continuous outcomes, we will use linear regression models to study quantitative outcome variables such as number of days respondent drank alcohol in the past 60 days (at baseline and 60-day follow-up).

**Analysis of Treatment Branches:** For analysis of within-treatment branches, we will use McNemar’s Test, which is a chi-square test used when data consist of paired observations on a particular outcome (i.e., pre-post treatment design), such as HIV disclosure with a new sex partner. For analysis of between-treatment branches, we will test differences across interventions or group mean differences by employing linear regression with interaction terms. We will also use ANOVA to examine within- and across-branch differences between those who complete the intervention activities and those who do not.

**Safeguarding against Confounding:** To safeguard against confounding in the multivariate logistic regression models, variables not significantly associated with outcomes in simple bivariate analyses may be included for conventional demographic items or if it is highly plausible (either biologically or behaviorally) that they are associated with the outcome.

**Interactions:** Tests for interactions will be conducted between pairs of explanatory factors and binary outcomes to assess possible interactions.

**Data Collection Procedures**

As participants complete each section of the online survey, the data will immediately be transferred to a Public Health Solutions designated server that cannot be accessed publicly. At the completion of data collection, an analytic file will be prepared by the Manager of Web Development and given to the data analyst in the Research and Evaluation Unit. The survey file, which has no personal identifiers, will be removed from the server and stored in a secure site. Only the study investigators and CDC collaborators will have access to the data. Because the analytic file has no personal identifiers, a CD will be kept in a locked file in the PI’s office for up to 10 years.

**Protection of Privacy/Confidentiality**

Participants will be assigned a unique study number. Behavioral survey (baseline, immediate post-intervention, 60-day follow-up) data will only include this unique study number. Email addresses of participants who agree to provide this information will be stored in an electronic file separate from survey data on a secure, firewall protected Public Health Solutions-managed server. The electronic file will contain email addresses linked to a participant’s unique study number. This linking file is necessary to identify and contact participants for the 60-day follow-up survey and track the result (i.e., completed follow-up or not). This file will be destroyed after the data collection period (i.e., 60-day follow-up assessments have been completed). Only the Public Health Solutions Manager of Web Development and Programming will have access to these files. Even in the highly unlikely event of a breach of the security system, the survey data exist only as a string of numbers with a few qualitative responses. The analytic data file has no personal identifiers and access will be limited to study staff.

**Lack of Need for Assurance or Certificate of Confidentiality**

We do not anticipate that any formal confidentiality protections will be needed for this research. While the behavioral assessments will include responding to some sensitive questions related to sexual behavior, sexual orientation, and drug use, participants’ names will not be collected. In conducting previous research with similar target populations, we have not observed that individuals have expressed reluctance to participate unless we are able to promise confidentiality. We also have no reason to doubt the validity of responses without the expectation of complete confidentiality.

**Information Management and Analysis Software**

The Web Development and Programming staff will program the surveys and electronic consent forms, and program computerized randomization for consenting intervention participants. Completed baseline and follow-up surveys will be downloaded into a SQL database, and then cleaned for missing data and incomplete cases. Statistical Software for the Social Sciences (SPSS) Version 14.0 and SAS 9.1 will be used for data cleaning and analysis.

**Data Management**

Public Health Solutions will utilize a web-based data monitoring tool to track recruitment and survey completion. There will be no way to identify participants using this tracking tool. Public Health Solutions will regularly report tracking tallies (with no person-level identifiable data) as the data collection period progresses. The assessment data will be encrypted and transferred routinely to CDC using a secure data network system. As in all of our other online studies, after data collection, we will thoroughly clean and conduct contingency checks on the data to ensure high quality of data, in terms of completeness and accuracy. We will report on the overall completion rate for the intervention, as well as the different steps in the consent process. The data will be released in SPSS and SAS formats to other investigators, although the data can be converted to other statistical software packages, such as STATA. We may require a data-use agreement with interested parties, depending on the variables being requested and whether the requested data have already been published or not. After cleaning and analyzing the data, we will report the strengths and limitations of the dataset, in terms of recommendations for data analysis, reporting, and interpretation.

**Quality Assurance**

After data collection, data will be checked for reliability and validity of value entries, and consistency across data elements. Since the provision of an email address is a requirement for participation in the intervention study, it is unlikely that duplicate records will be an issue for this study. However, the study team will be unable to identify if an individual has enrolled into the study more than once using differing email addresses. Should this occur the duplicative data will likely remain in the data file. This occurrence is likely to be infrequent, considering the lack of a large incentive. All quality assurance measures taken during data collection will be documented and made available to collaborators. Finally, to guard against bias in the analysis and interpretation of the data, analyses will be performed and reviewed by both scientists at Public Health Solutions and CDC.

**Bias in Data Collection, Measurement, and Analysis**

In order to eliminate or reduce possible bias in the proposed intervention, we will be programming the baseline survey to select and randomly assign participants who consent to be part of the study. There will be no human involvement in the process of selecting and assigning participants. Participants will not be blinded once they are randomized to their treatment or control condition, however they will be told very little about the other possible conditions (See Appendix C for the consents). This will minimize multiple attempts to enroll in the study in order to achieve a different assignment. It is still possible that a participant could attempt to complete the baseline survey several times; however, given the length of the survey and absence of incentives this is unlikely.

**Intermediate Reviews and Analysis**

During the data collection phase, we will conduct random checks of the data to ensure proper collection into the SQL database.

**Response to New and Unexpected Findings/Changes in Environment**

If during data collection there is a significant external event (e.g., the announcement of a fast-spreading, multi-drug-resistant HIV strain), we can immediately respond by altering the survey online to ask additional questions in response to this event.

**Limitations of Study**

There are several limitations to consider in this study.

1. Minority MSM may be under-represented in this sample; however the study team will monitor this during the course of enrollment. It may be necessary to increase the projected enrollment figures in order to achieve an adequate sample of this population. Any adjustments in enrollment figures will be submitted as an amendment to local and CDC IRBs.
2. Similar to mail-in, phone, and other non-face-to-face survey research, the study team will be largely unable to verify the identity of participants or the reliability of responses to the survey instruments.
3. It is not possible for the study team to determine whether this study population is representative of MSM using the internet, or MSM in general given that this population has never been enumerated.

**Anticipated Products/Interventions and Use**

Findings from this intervention can be used to help guide web-based HIV prevention efforts. Further, the information from this intervention has tangible benefits to drug prevention and sexual risk reduction efforts nationally. Information from this intervention will better inform public health agencies about the magnitude of these behaviors and help them to develop outreach and prevention strategies that address the needs of MSM who use the Internet from their states. The end-product of the work will be a standardized protocol ready for internet-based intervention trials to have significant reach among MSM online.

**Notifying Participants of Study Findings**

There will be a message at the end of the survey, notifying participants that the study findings will be available on the Public Health Solutions website in the coming year. The Public Health Solutions website address will be listed.

**Dissemination of Results to Public**

Findings will be disseminated at domestic and international scientific meetings and through peer-reviewed publications. We will also disseminate our research findings via local and national media and make study findings available for participants and other interested individuals as an online downloadable report on Public Health Solutions website. Pulse Studies (synopses of previous online and offline Public Health Solutions studies) are available on the Public Health Solutions website and are distributed to more than 1,000 elected officials, public health professionals, policy makers, service providers, advocates and media outlets. Findings will also be presented to the community via discussion groups and forums.

# IV. REFERENCES

**APPENDIX A.**

**ONLINE INTERVENTION PILOT / FORMATIVE WORK**

In October 2005, MHRA, in collaboration with New York University (Dr. Francine Shuchat Shaw), conducted a national online behavioral intervention pilot on Manhunt.net. An 8-minute dramatic video, **The Morning After**, was designed, written and filmed to promote critical thinking about HIV disclosure, risky sexual behaviors and the complex beliefs and attitudes underlying these behaviors. For this proposed project, The Morning After dramatic video will serve as an intervention for sexual risk reduction within the context of alcohol use.

**Dramatic Video, The Morning After:** The approach we used for this video is grounded in traditional social cognitive theory, which has been successful in many other educational settings. To the best of our knowledge, we are the first to show that it is also successful as an online intervention pilot. The evaluation of our dramatic video was completed in February 2006. MSM were recruited through banner ads on the exit page of Manhunt.net. Over 1,000 men from 48 of 50 states completed a baseline behavioral survey, watched the video, completed the post-video survey, and provided email addresses for a 3 month follow-up.

**Description of The Morning After:** Directed by Todd Ahlberg, this pilot video vignette was produced for the HIV Is Still A Big Deal Project (MHRA & NYU, 2005). This 8-minute dramatic video is a fictional story with carefully crafted plot and characters based on reality. This vignette tells the story of four gay friends, and focuses on two of them. Josh awakens the morning after excessively drinking and hooking-up with Eric to find antiretroviral medications in Eric’s bathroom. He quickly finds his friends to talk about this, after which Eric arrives to talk with Josh as well. In the course of this short drama, issues of disclosure (“asking” and “telling”), alcohol use, condom use, and HIV testing are expressed in the situation and the conversation.

***Title: Is HIV Still a Big Deal?***

***Baseline***

M.A. Chiasson, PI

Between October 11 and 22, 2005 we conducted our first national online behavioral intervention on Manhunt.net (funding from the van Ameringen Foundation, The New York Community Trust, and The Shelley and Donald Rubin Foundation). **The Morning After** was designed to promote critical thinking about HIV disclosure, risky sexual behaviors and the complex beliefs and attitudes underlying these behaviors. Approximately 13,500 men clicked on the online banner ad, and out of those, 3,139 consented to participate in the intervention; 2,777 completed the baseline behavioral risk survey (pre-test); 1601/2777 watched the video about a sexual encounter between an HIV-positive and HIV-negative male; 1510/1601 completed the questions about HIV testing and disclosure after the video (post-test); and 1087/1510 consented to give us their email address for a follow-up behavioral risk survey in 3 months. Online, 1,002 men completed the baseline behavioral survey, watched the video, completed the post-video survey and provided valid email addresses for 3 month follow-up. The median age of men consenting to participate was 38.

***Follow-up*: Validation of the Online Video as an Intervention**

Of the 1,002 men who completed the survey and provided emails, 3 months later, 908 emails were still working. Emails linking to the follow-up survey were sent 1/17, 1/23 and 2/1/06 to 908/1,002. No incentives were provided. Preliminary analysis compared disclosure of HIV status to sexual partners and HIV testing, before and in the 3 months after watching the video, by McNemar and Pearson Chi-square tests. Three months after watching the video, 496 of 908 men (55%) with working emails completed the follow-up survey. Men completing follow-up and those who did not were similar by race/ethnicity (14% Hispanic, 72% white, 6% black, 2% Asian, 6% mixed/other), age (mean 38) and prevalence of unprotected anal intercourse with a new/casual partner (36%), but were significantly more likely to have a college degree (55% vs. 47%, p=.01). Statistically significant behavior changes were found. Men were 3 times more likely to ask their partner’s HIV status (OR=3.0, p<.001) and two times more likely to disclose their own (OR=2.2, p<.001) at follow-up compared to baseline. The 318 HIV-negative men participating in the evaluation were also three times more likely to report HIV testing during follow-up (42%) than in the 3 months before baseline (20%) (OR=2.8, p<.001). These findings strongly suggest that online interventions designed to engage critical thinking are an efficient and effective way to influence behavior change in large numbers of at-risk MSM.

We examined the prevalence and stability of drug and alcohol use between baseline and follow-up. At baseline, 7% reported methamphetamine use in their last sexual encounter. At follow-up, 5% reported methamphetamine in their last encounter. Out of the methamphetamine users at baseline, 39% followed up and were significantly LESS likely than non-users to follow-up (OR 0.6, p<.05). Contrary to methamphetamine use, other drug use (i.e., poppers, marijuana, MDMA, GHB, cocaine) as well as alcohol use, reported between baseline and follow-up remained stable. Specifically, there were no significant differences in the proportions of men reporting drug or alcohol use at baseline compared to follow-up. Drug use at baseline and follow-up was 24% and 32%; alcohol use was 27% at both baseline and follow-up.

**ONLINE RESEARCH STUDIES**

***Study 1:* Web-based Survey of Sexual Behavior among Gay and Bisexual Populations**

M.A. Chiasson, PI

Between June 3 and July 24, 2002 an anonymous, cross-sectional online survey was conducted, inquiring about general sexual and drug-using behaviors among MSM during a recent 6-month period. The survey was displayed in the chat room area of a gay-oriented website.^24, 54, 55^ Approximately 6,000 participants clicked on the banner; 2,284 exited the survey without answering any questions; 2,915 men who completed the survey reported sex with other men or self-identified as gay or bisexual. Participants resided in all fifty states. The sample was predominately white (85%). Nearly half of the sample was between 18 and 29 years of age (range 18-60+). Most reported up to $40,000 income and at least some college education or more. Most men (80%) reported having sex only with men. Overall, 8% of the sample reported testing HIV-positive. During the six-month study period 80% reported more than one sex partner. Forty-five percent reported any illicit drug use and about half reported drinking alcohol before or during sex. Approximately 68% of those reporting drug use reported drugs before or during sex. The self-reported HIV prevalence by age group (which included men in the denominator who were not tested during the 6-month period the study asked about) was 2% for 18-24, 6% for 25-29, 12% for 30-39, 10% for 40-49, and 13% for 50 or older.

***Study 2: Behavioral Risk in Men Recruited Online***

M.A. Chiasson, PI (CDC Overall study); S. Hirshfield, PI (R03, NIDA)

Between October 2003 and March 2004, we conducted our second anonymous, cross-sectional Internet study (funded by the CDC, 200-97-0621, Task 33 to RTI Intl.; R03 funded by NIDA, DA018725-01), inquiring about sexual and drug-using behaviors in a specific sexual encounter among MSM.^25^ Men were recruited online via a banner linking to the survey, which was posted on 14 different gay-oriented websites. Over 4,000 men completed the survey. Among men reporting sex with new/casual partners within the past 3 months (N=1,683), the median age was 36 (range 18-85); 80% were white; 51% had college degrees; 52% earned $40,000 or more; and 87% reported sex with only men. In their last encounter, 48% met their last sex partner online; 24% reported drugs and 28% reported alcohol before/during sex. Specific drugs reported before/during sex included marijuana (10%), poppers (7%), Viagra (7%), and crystal methamphetamine (4%). Approximately 23% reported two or more sex partners in the last encounter. HIV prevalence by age group (which included men in the denominator who reported HIV testing results, N=1,298) was 3% for 18-24, 7% for 25-29, 14% for 30-39, 16% for 40-49, and 7% for 50 or older.

***Study 3: Viagra, Drug Use and HIV Transmission in Men Who Have Sex with Men***

S. Hirshfield, PI

Between November 2004 and March 2005, we conducted our third anonymous, cross-sectional online study (funded by an independent medical grant from Pfizer, Inc.), inquiring about substance use and sexual risk behaviors with and without pde-5 inhibitors (i.e., Viagra, Levitra, or Cialis) within the past 12 months. Over 19,000 respondents clicked on the consent form, and approximately 11,300 cases were complete enough for statistical analysis. Eight websites hosted banners linking to an anonymous questionnaire, which asked about behavior in the past 12 months. 7,764 men from the US or Canada reported sex with men or both men and women within 12 months prior to the survey. The median age was 38 (range 18-85); 82% were white; 63% earned $40,000 or more; 93% reported sex with only men; 31% reported having a main partner; 61% of men without a main partner met their last sex partner online; 23% reported drugs and 22% reported alcohol before/during sex. Specific drugs reported before/during sex included marijuana (8%), poppers (10%), Viagra/Levitra/Cialis (13%), and crystal methamphetamine (8%). Approximately 14% reported two or more partners in the last encounter. HIV prevalence by age group (which included men in the denominator who reported HIV testing results, N=6,465) was 4% for 18-24, 6% for 25-29, 14% for 30-39, 22% for 40-49, and 19% for 50 or older.

**APPENDIX B. Survey Banner**


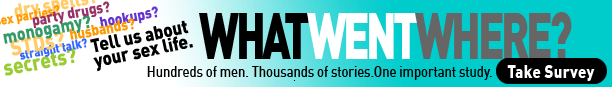


**Appendix C. Informed Consents**

**C-1. CONSENT TO PARTICIPATE IN A SCIENTIFIC RESEARCH STUDY (Flesch-Kincaid=7.8)**

**Introduction**

To better understand the sexual practices of men, we need your help. We are asking you to join a research study. The study will help us provide health information about safer sex and prevention of HIV or other sexually transmitted infections.

Public Health Solutions (formerly known as Medical and Health Research Association of New York City) and the Centers for Disease Control and Prevention are conducting this study. We plan to enroll around 10,500 men to take this survey.

**Study Activities**

This study is for men who are age 18 or older, have had sex with another man, and who live in the United States. If you decide to join the study, we will ask you to take a survey. The survey will take about 10-15 minutes. We will ask you questions about:

- your sexual practices,
- your use of drugs and alcohol,
- your health, and
- HIV testing (HIV is the virus that causes AIDS)

After the survey, you may also be eligible to take part in other study activities. If you decide to participate in those activities, you will either view one of two brief videos, both videos, a website that contains HIV prevention information, or do nothing. These activities will take between 1 and 20 minutes, depending upon the assignment. Also, we will ask you a few more questions about things you plan to do in the next 60 days. After 60 days, we will invite you to complete a third and final online survey. If you are eligible for these activities, we will explain them in more detail after the first survey. You can decide then if you want to join.

**Privacy**

You will be assigned a unique study identification number. We will not ask your name. We will not use “cookies” to track any personal information. We will not record the IP address of your computer. This means that information you give cannot be traced to your computer or to you. Your survey responses will be combined with the responses of other men when we present this study or publish results. Therefore, nothing that you say will be traced back to you. All of the study data will be stored on a secure password protected computer system at Public Health Solutions.

**Risks**

The only known risk to you for taking the survey is potential embarrassment or anxiety. Some survey questions are personal and sensitive. You can refuse to answer any question or decide to not take the survey. The chance of a break of confidentiality is minimal due to the data security procedures at Public Health Solutions, such as password protection and a firewall system.

**Benefits**

After the survey, you can follow links to information about how to prevent sexually transmitted diseases and other health related issues. Also, your input will help us to improve how we provide health information to other men like yourself.

**Costs and Payments**

There is no cost to you for joining the study. You will not be reimbursed for taking this survey.

**Voluntary Participation**

Joining this study is voluntary. You can change your mind and decide not to take part at any time. If you do not want to join, click the NO button below or close your browser window. Declining to join the study will not affect your use of the website you are on.

**Persons to Contact**

If you have questions about your rights as a participant, you may call Dr. T. Rosenberg, Administrator of the Public Health Solutions IRB at (646) 619-6680 or [trosenberg@healthsolutions.org](mailto:trosenberg@healthsolutions.org). If you have questions about the study, you may contact Dr. S. Hirshfield, Principal Investigator of the study at (646) 619-6676 or [surveyquestions@healthsolutions.org](mailto:surveyquestions@healthsolutions.org).

CONSENT STATEMENT:

I have read this form and have decided to join this study.

[
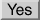
](http://ostinato.stanford.edu/coping/survey.asp)[
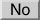
](http://ostinato.stanford.edu/coping/nothanks.html) **Click here to submit this form.**

**C-2. CONSENT TO PARTICIPATE IN INTERVENTION STUDY AND GIVE E-MAIL ADDRESS FOR FOLLOW-UP SURVEY (Flesch-Kincaid=7.9)**

**Introduction**

Thank you for your participation. Now, we are inviting you to join another study activity related to the survey you just completed. This is also being done by Public Health Solutions (formerly Medical and Health Research Association of New York City) and the Centers for Disease Control and Prevention. We plan to enroll around 3,000 men for this part of the study.

**Study Activities**

If you decide to join this part of the study, the following will happen:

(1) A computer program will automatically “randomize” you into 1 of 5 study groups. This means that you will be placed into a study group by chance. It is like flipping a coin. You will have a 1 in 5 chance of being placed in any group. You will either view one of two brief videos, both videos, a website that contains HIV prevention information, or do nothing. These activities will take between 1 and 20 minutes, depending upon the assignment.

(2) We will ask you a few more questions about things you plan to do in the next 60 days. This should take less than a minute.

(3) You will have the option of viewing a resource page with links to health information. We will note which links you choose. We will use this information to better understand what types of services men like you are interested in accessing.

(4) After 60 days, we will invite you to complete a third and final online survey. This survey is like the one you did today.

To join this study, we will need your e-mail address. We will use your e-mail address to contact you for the final survey. We will not ask your name or any other identifiable information. Below there is a space for you to provide your email address. We will send you an e-mail reminder 1 week before the final survey. The email will contain a link to the survey.

**Privacy**

We will not ask your name. Your e-mail address will be the only identifiable information collected, and it will be stored in our protected computer system in an unreadable format. It will be kept separate from your survey responses, but linked to your unique study identification number. Your email address will automatically be deleted from our system after the study ends.

We will not use “cookies” to track any personal information. We will not record the IP address of your computer. This means that information you give cannot be traced to your computer or to you. Your survey responses will be combined with the responses of other men in the study. Therefore, nothing that you say will be traced back to you. All of the study data will be stored on a secure password protected computer system at Public Health Solutions.

**Risks**

There is minimal risk for being in this study. One known risk is potential embarrassment or anxiety when answering some questions on the survey, which are personal and sensitive. You can refuse to answer any question. You can also decide not to take the survey or view study materials. The chance of a break of confidentiality is minimal due to the data security procedures at Public Health Solutions, such as password protection, email address encryption, and a firewall system.

**Benefits**

After the survey, you can follow links to information about how to prevent sexually transmitted diseases and other health related issues. Also, your input will help us to improve how we provide health information to other men like yourself.

**Costs and Payments**

There is no cost to you to be in the study. You will not be reimbursed for taking this survey.

**Voluntary Participation**

Taking part in this study is completely voluntary. If you do not want to join, click the NO button. If you decide to stop the survey at any point, just close your browser window. Declining to join the study will not affect your use of the website you are on.

**Persons to Contact**

If you have questions about your rights as a participant, you may call Dr. T. Rosenberg, Administrator of the Public Health Solutions IRB at (646) 619-6680 or [trosenberg@healthsolutions.org](mailto:trosenberg@healthsolutions.org). If you have questions about the study, you may contact Dr. S. Hirshfield, Principal Investigator of the study at (646) 619-6676 or [surveyquestions@healthsolutions.org](mailto:surveyquestions@healthsolutions.org).

CONSENT STATEMENT:

I have read this form and have decided to join this study. This involves providing my email address, being randomly assigned to a group, doing a short survey after the assigned activity, and completing a follow-up survey in two months.

**Type email address here**

**Re-type email address here**

[
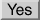
](http://ostinato.stanford.edu/coping/survey.asp)[
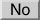
](http://ostinato.stanford.edu/coping/nothanks.html) **Click here to submit this form.**

**C-3. REMINDER ABOUT YOUR STUDY RIGHTS AS A PARTICIPANT (Flesch-Kincaid=8.9)**

**Introduction**

Sixty days ago, you agreed to join a study about sexual practices of men and completed a survey about your sexual behavior. You gave us your email address to contact you. Public Health Solutions (formerly Medical and Health Research Association of New York City) and the Centers for Disease Control and Prevention are doing the study. Your answers will help us learn more about men and their sexual behaviors. It will help us provide health information about safer sex and how to prevent HIV or other sexually transmitted infections.

**Study Activities**

We are asking you to complete a final survey that is similar to the previous one. The survey will take about 10 minutes. We will ask you questions about

- your sexual practices,
- your drug and alcohol use,
- your health, and
- HIV testing.

After the survey, you will be shown a resource page with links to health information. We will note which links you choose. We will use this information to better understand what types of services men like you are interested in accessing.

**Privacy**

Your e-mail address was stored in our protected computer system in an unreadable format. It was kept separate from your survey responses, but linked by a unique study identification number. Your email address will automatically be deleted from our system after the study ends.

We will not ask your name or any other identifiable information. We will not record the IP address of your computer. Your survey responses will be combined with the responses of other men. Therefore, nothing that you say will be traced back to you.

**Risks**

One known risk to you for taking part in the research project is potential embarrassment or anxiety. Some questions on the survey are personal and sexually explicit. You can refuse to answer any question. You can also decide to not take the survey. The chance of a break of confidentiality is minimal due to the data security procedures at Public Health Solutions, such as password protection, email address encryption, and a firewall system.

**Costs and Payments**

There is no cost to you to be in the study. You will not be reimbursed for taking this survey.

**Voluntary Participation**

Taking part in this study is completely voluntary. If you decide to stop the survey at any point, just close your browser window. Declining to complete the last survey will not affect your use of the website you are on.

**Persons to Contact**

If you have questions about your rights as a participant, you may call Dr. T. Rosenberg, Administrator of the Public Health Solutions IRB at (646) 619-6680 or [trosenberg@healthsolutions.org](mailto:trosenberg@healthsolutions.org). If you have questions about the study, you may contact Dr. S. Hirshfield, Principal Investigator of the study at (646) 619-6676 or [surveyquestions@healthsolutions.org](mailto:surveyquestions@healthsolutions.org).

**CLICK HERE TO GO TO NEXT PAGE (START THE SURVEY)**

**CLICKE HERE TO CLOSE YOUR BROWSWER (DO NOT START THE SURVEY)**

**PRINT**

**APPENDIX D. DESCRIPTION OF ASSIGNED BRANCH CONDITIONS**

**Branch 1** (control): “We will now ask you 6 brief questions.”

**Branch 2** (standard text-based prevention webpage) http://www.cdc.gov/hiv/topics/msm/index.htm:

“Now you will see a text-based HIV prevention website. When you are finished, you will be asked 6 short questions.”

**Branch 3** (Morning After video):

“Now you will see an 8-minute dramatic video about four gay friends. When you are finished, you will be asked 6 short questions.”

**Branch 4** (Talking About HIV video):

“Now you will see a 5-minute documentary video about gay men. When you are finished, you will be asked 6 short questions.”

**Branch 5** (both videos):

“Now you will see 2 brief videos (totaling 13 minutes) about gay men. When you are finished, you will be asked 6 short questions.”

**APPENDIX E. BASELINE / FOLLOW-UP SURVEY (same general survey for both time points with the following exceptions to the follow-up survey described below)**

Please see pdf document (Hirshfield Intervention Survey)

**The following items are included in the baseline survey but excluded from the follow-up:**

I am a... (Gender)

How would you describe your sexual orientation?

Where do you live?

The city that I live in has...

My annual income is:

Are you currently employed?

How many times have you moved in the past year?

Have you ever had oral or anal sex with a male?

Have you ever had oral, vaginal, or anal sex with a female?

Past year / Lifetime Sexual history questions for males and females

About how old were you when you first had a drink?

In your lifetime: have you ever injected...

In your lifetime: have you ever had a blood transfusion?

In your lifetime: have you ever been tested for Hepatitis B?

In your lifetime: have you ever been vaccinated for Hepatitis B?

In your lifetime: have you ever been tested for Hepatitis C?

How many times have you been tested for HIV?

Do you have a primary care provider?

Do you currently have health insurance?

Have you ever spent at least 1 night in jail or prison?

Which best describes your weight?

**The following items are included in the baseline survey but the time period will be modified to the past 60 days for the follow-up survey:**

In the past 60 days...

Have you been diagnosed with the following?

Have you had mental health counseling or treatment from a mental health provider? y/n

Have you attended any alcohol or drug treatment programs (such as twelve-step program)? y/n

(please specify)________

Have you seen an online video about HIV? (check all that apply)

□ Yes, in this study

□ Yes, on Youtube

□ Yes, on another website (please name the site)__________

Following completion of the baseline survey participants will be provided with the following instructions depending upon eligibility to continue further:

If ineligible, the participant will receive a message saying, “Thank you for your time. We appreciate your input in this study. Here are some links to helpful websites about staying healthy. Please check back on the MHRA website in 2008 for a description of the study’s results.”

If eligible, the participant will automatically be directed to the C-2 Consent Form.

**APPENDIX F. POST-INTERVENTION INTENTION QUESTIONS**

| **{ Post Intervention Questions}** | | |
| --- | --- | --- |
| In the next 60 days... | Definitely probably might probably definitely  will will won’t won’t | I prefer not to answer () |
| How likely are you to... |  |
| …ASK your new sex partners their HIV status? | () () () () () |
| …TELL your new sex partners your HIV status? | () () () () () |
| …use condoms with new sex partners? | () () () () () |
| …reduce alcohol use before sex? | () () () () () |
| …reduce drug use before sex? | () () () () () |
| {if negative or untested}  Do you plan to get an HIV test? | () () () () () |  |

“Thank you for participating in the study. In 60 days, we will contact you using the email address you provided to ask you some additional questions. Here are some links to helpful websites about staying healthy.”

**APPENDIX G. EXAMPLE OF SURVEY EXIT PAGE**

Please provide comments or ask questions here. You can use your email address or not.
